# Supplementary material for: General and specific patterns of cortical gene expression as spatial correlates of complex cognitive functioning
Source: bioRxiv. 2023 Sep 20:2023.03.16.532915. Originally published 2023 Mar 17. Preprint. [Version 2] doi: 10.1101/2023.03.16.532915 (PMC10055068; doi:10.1101/2023.03.16.532915)
Supplement: Supplement 1 [file media-1.pdf]

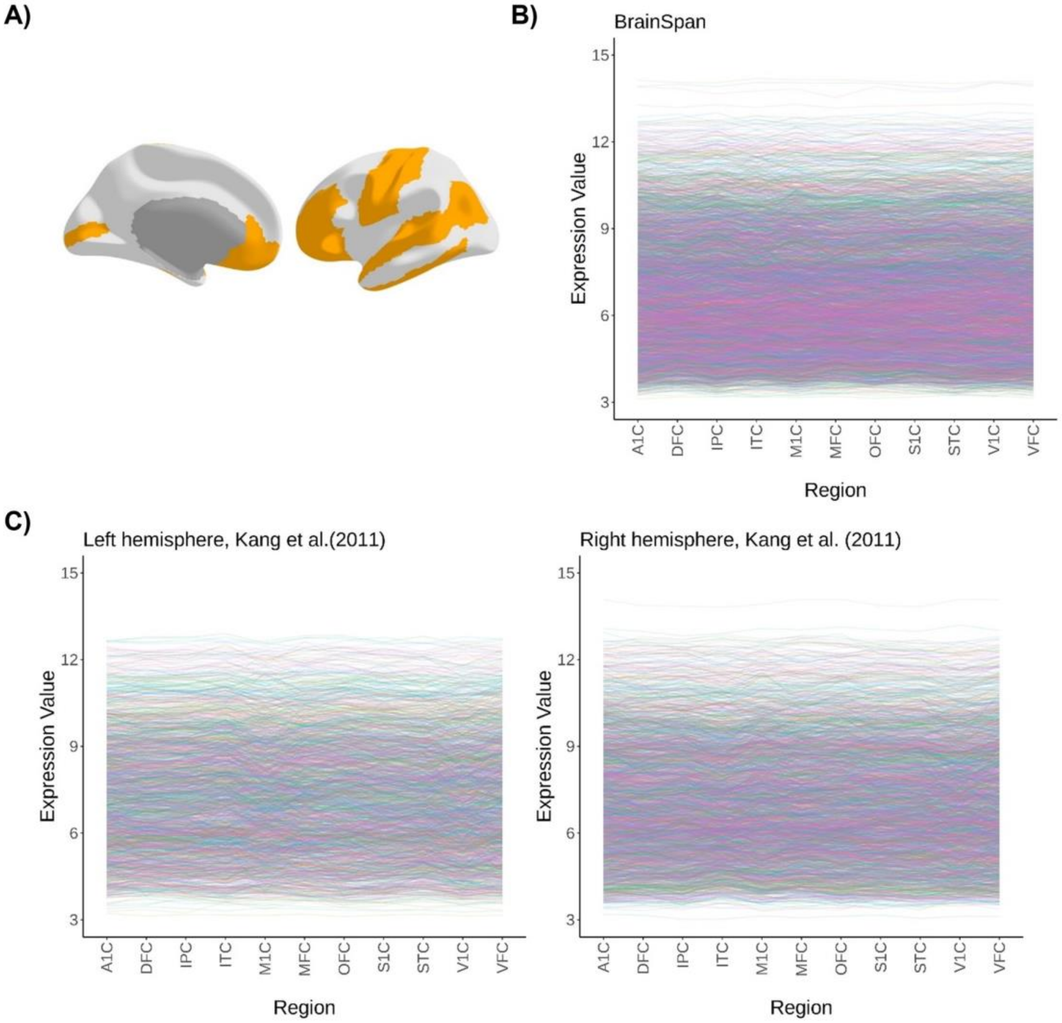

Figure S1 Raw gene expression values for the two validation datasets, for genes that have between-donor consistency and are matched with the 8235 consistent genes in the Allen dataset. A) The 11 cortical regions of the two validation datasets roughly matched to 14 Desikan-Killiany regions, as suggested by Wong et al.[1] B) Data from the Brainspan dataset. Bilateral regions, 4410 consistent and matched genes, and C) Gene expression data from Kang et al. (2011) for the left hemisphere, 2030 consistent and matched genes and for the right hemisphere, 2778 genes.

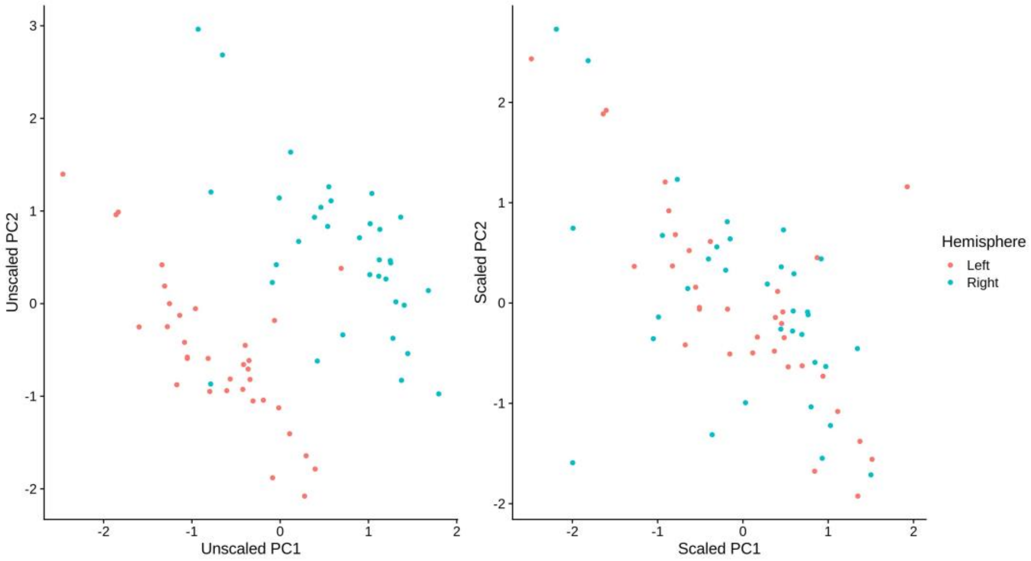

Figure S2 Principal component scores for Component 1 and Component 2, after varimax rotation. Left: before scaling the scores in each hemisphere; Right: after scaling each hemisphere.

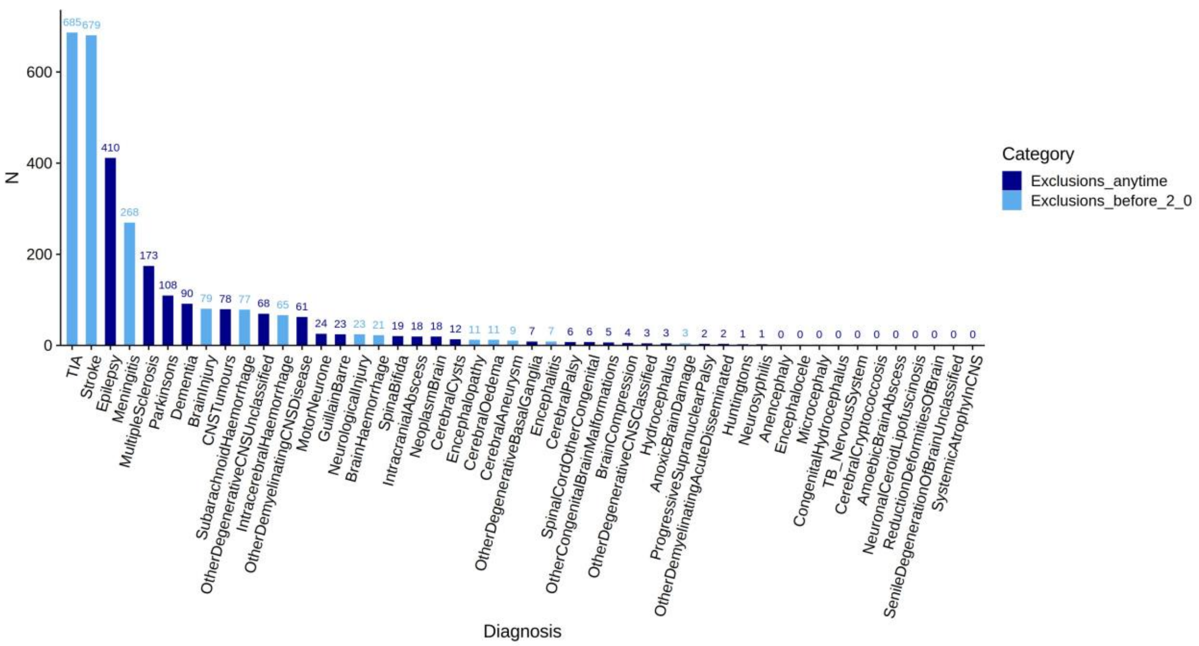

Figure S3 N exclusions by diagnosis in the UKB sample (total *N* of participants with any diagnosis = 2542). Initial UKB *N* (participants included in FreeSurfer bulk download) = 40,382 -> UKB *N* = 2542 excluded on medical grounds -> final UKB *N* = 37,840.

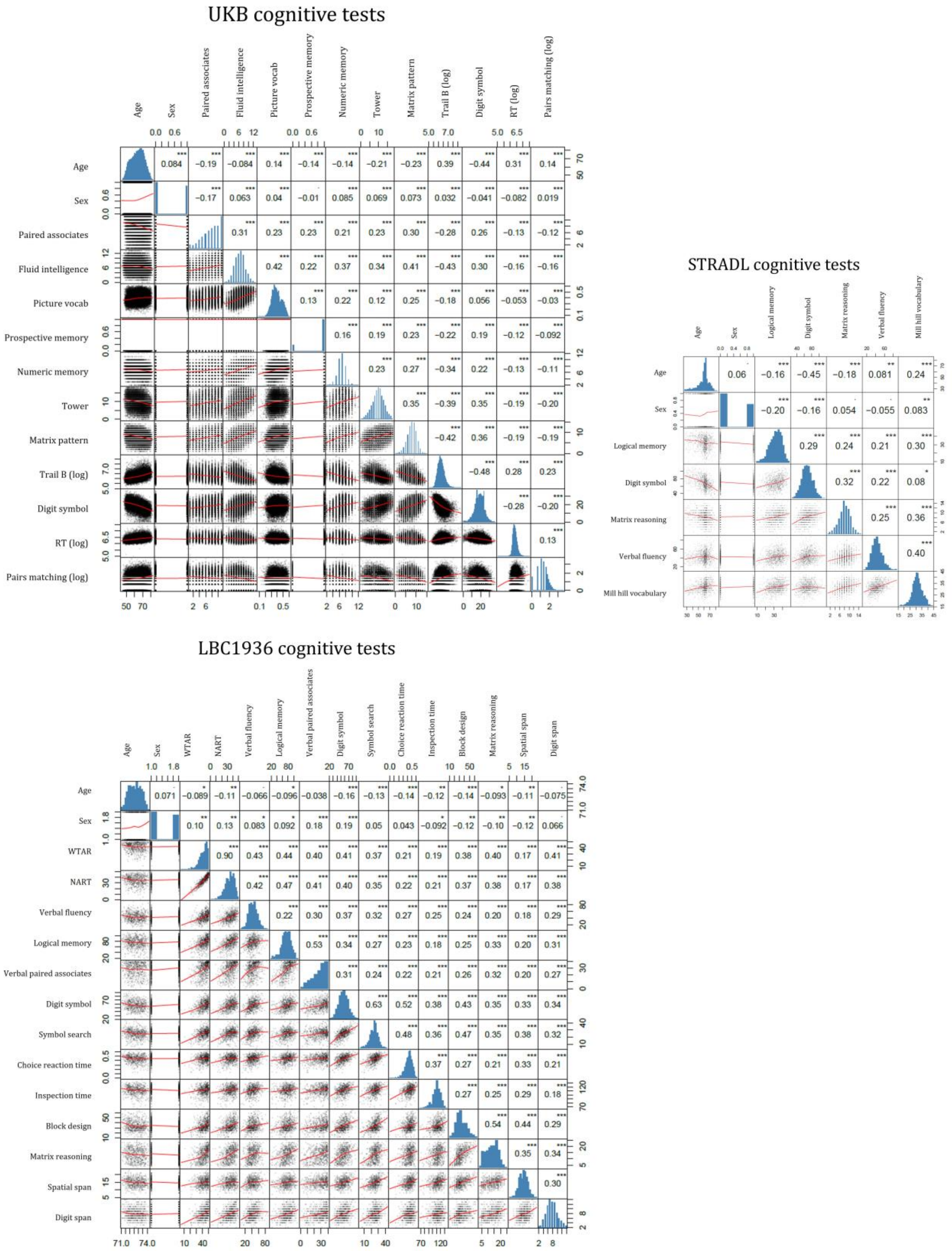

Figure S4 Correlation plots of the individual cognitive tests within each cohort, also showing data distributions, and correlations with age and sex.

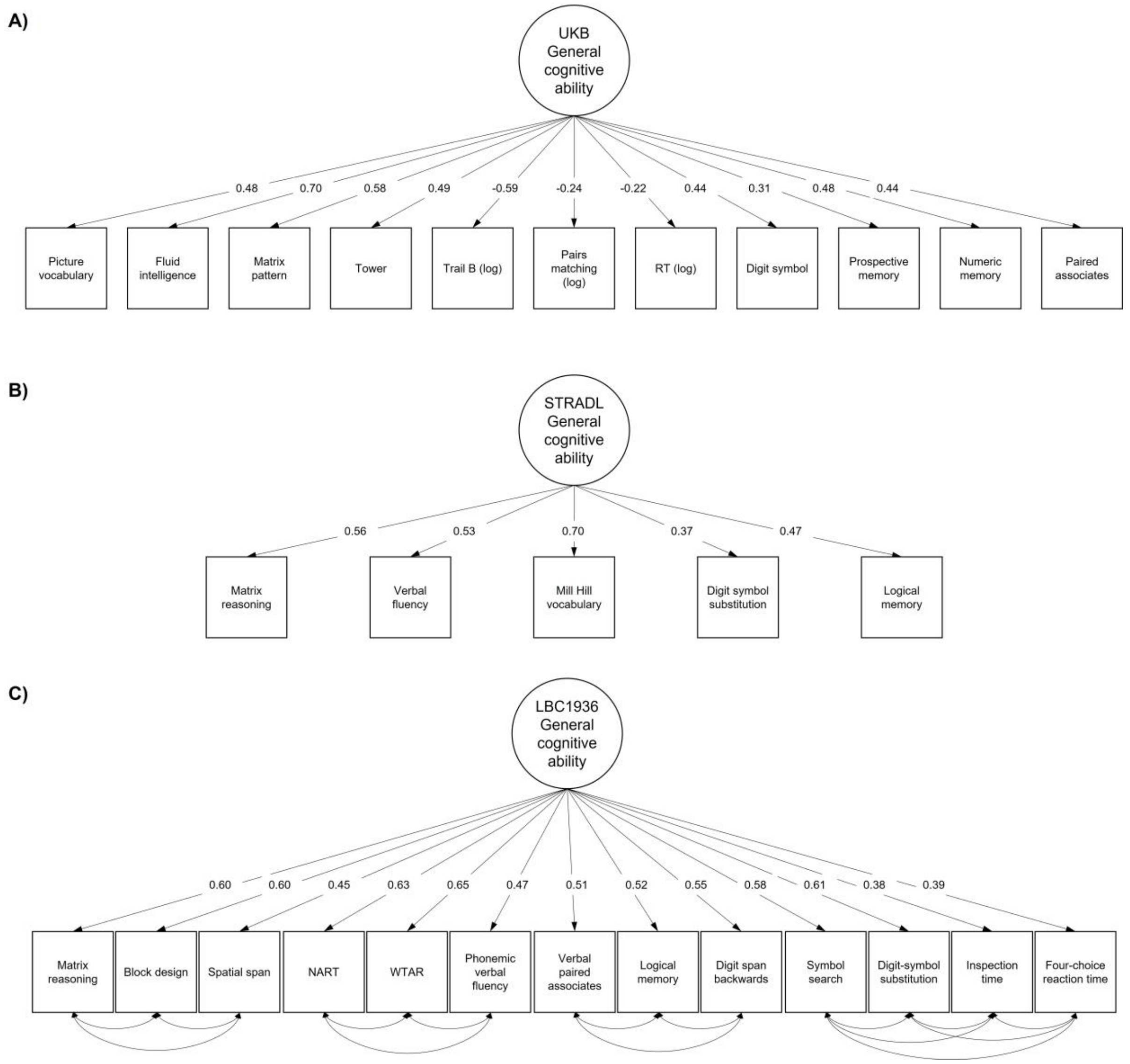

Figure S5 Simplified path diagrams of cognitive ability latent models alongside density plots of the final cognitive ability prediction *z* scores for A) UKB, B) STRADL, and C) LBC1936. The within-domain residual variances for LBC1936 are in *Table S*17. Model fits are in *Table S18*.

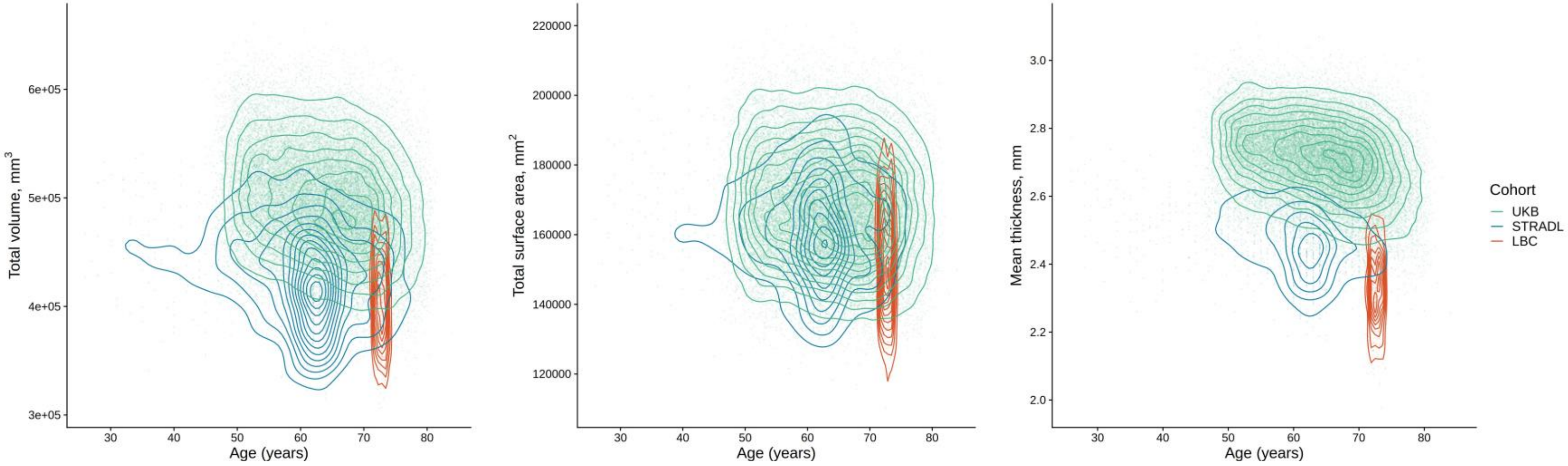

Figure S6 Total volume (mm3), total surface area (mm2), and mean thickness plotted by age for each cohort (UKB, STRADL and LBC).

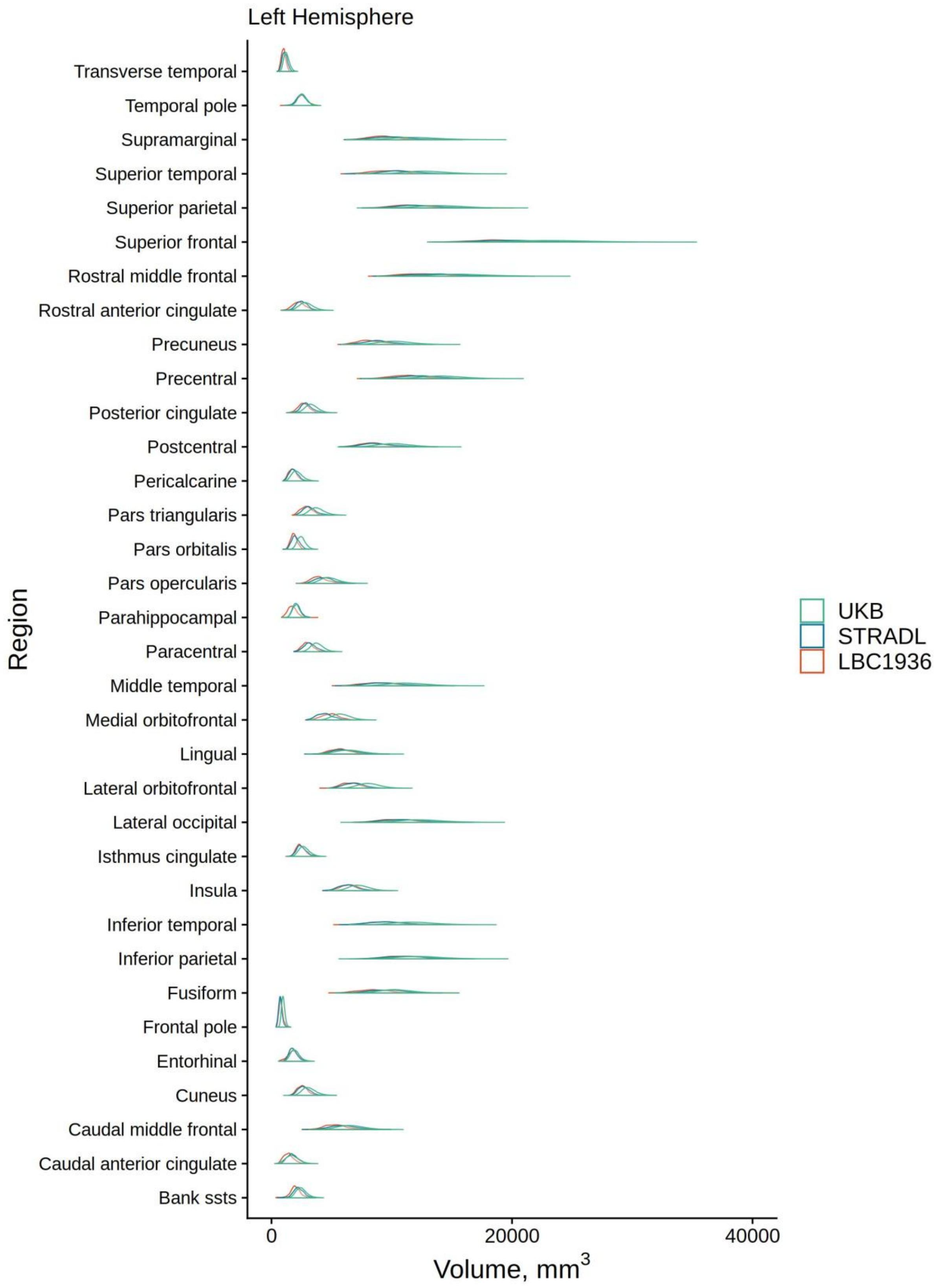

Figure S7 Regional density plots of left hemisphere volume (mm3), for UKB, STRADL and LBC1936 .

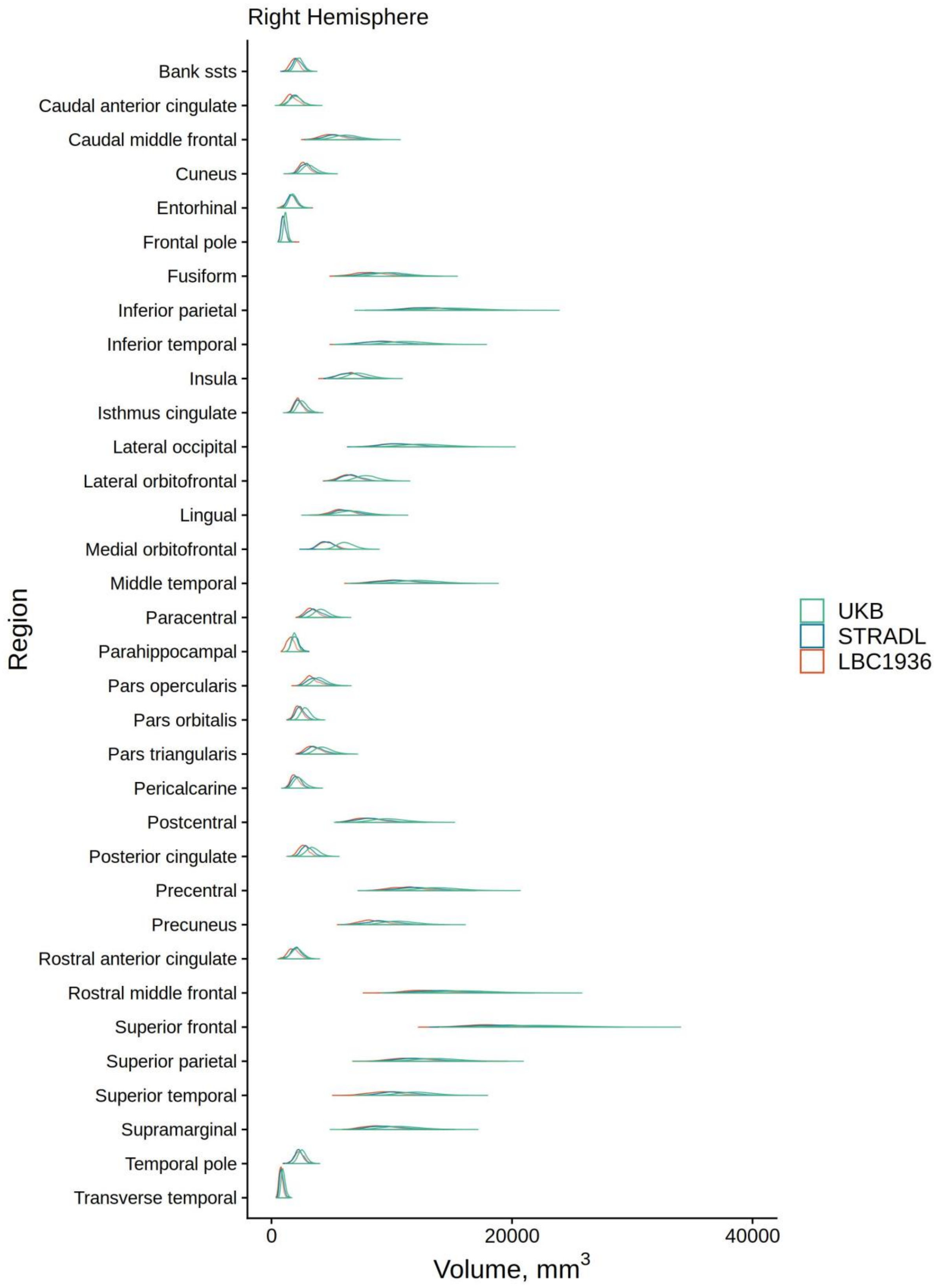

Figure S8 Regional density plots of right hemisphere volume (mm^3), for UKB, STRADL and LBC1936.

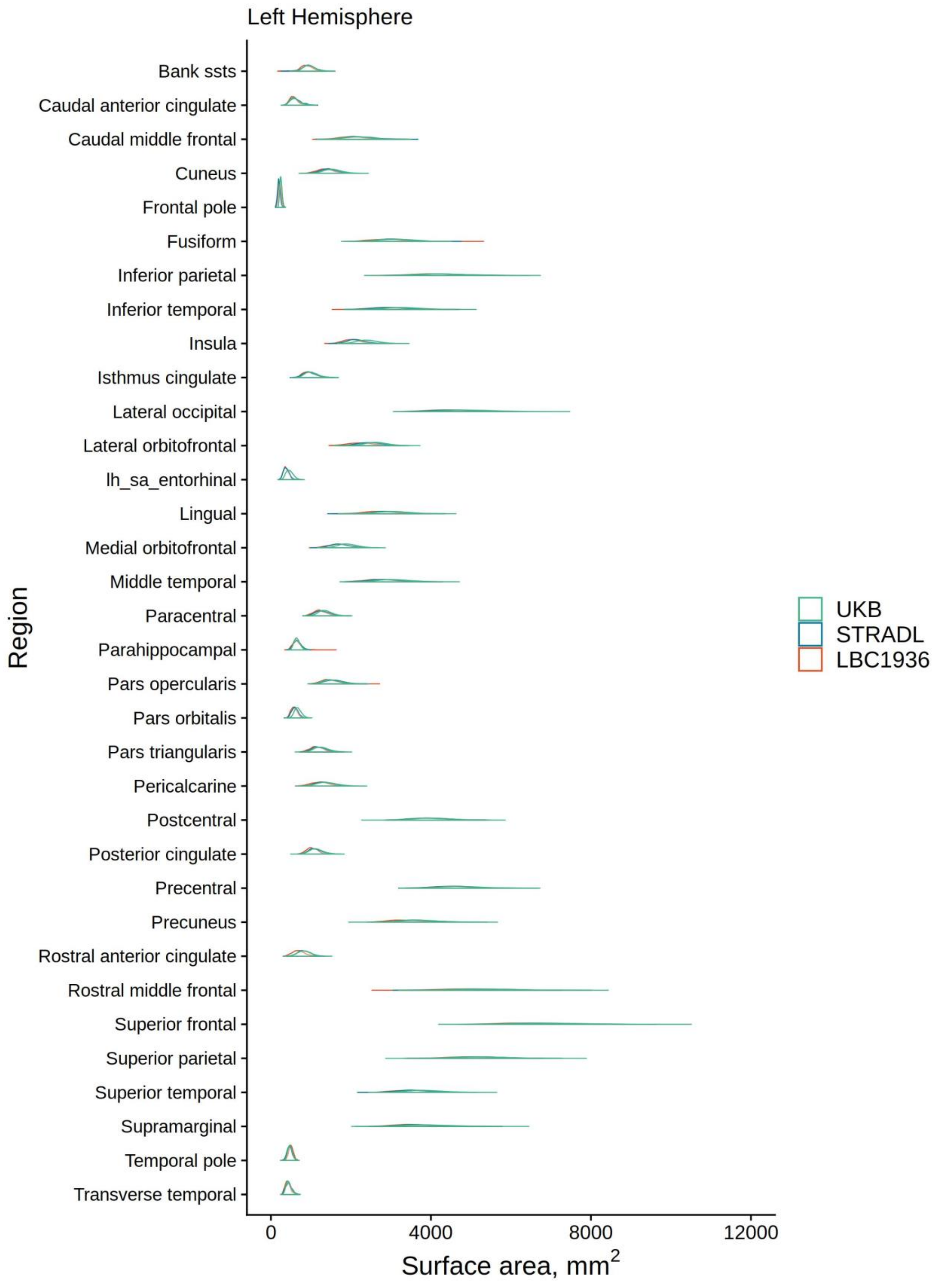

Figure S9 Regional density plots of left hemisphere surface area (mm^2), for UKB, STRADL and LBC1936.

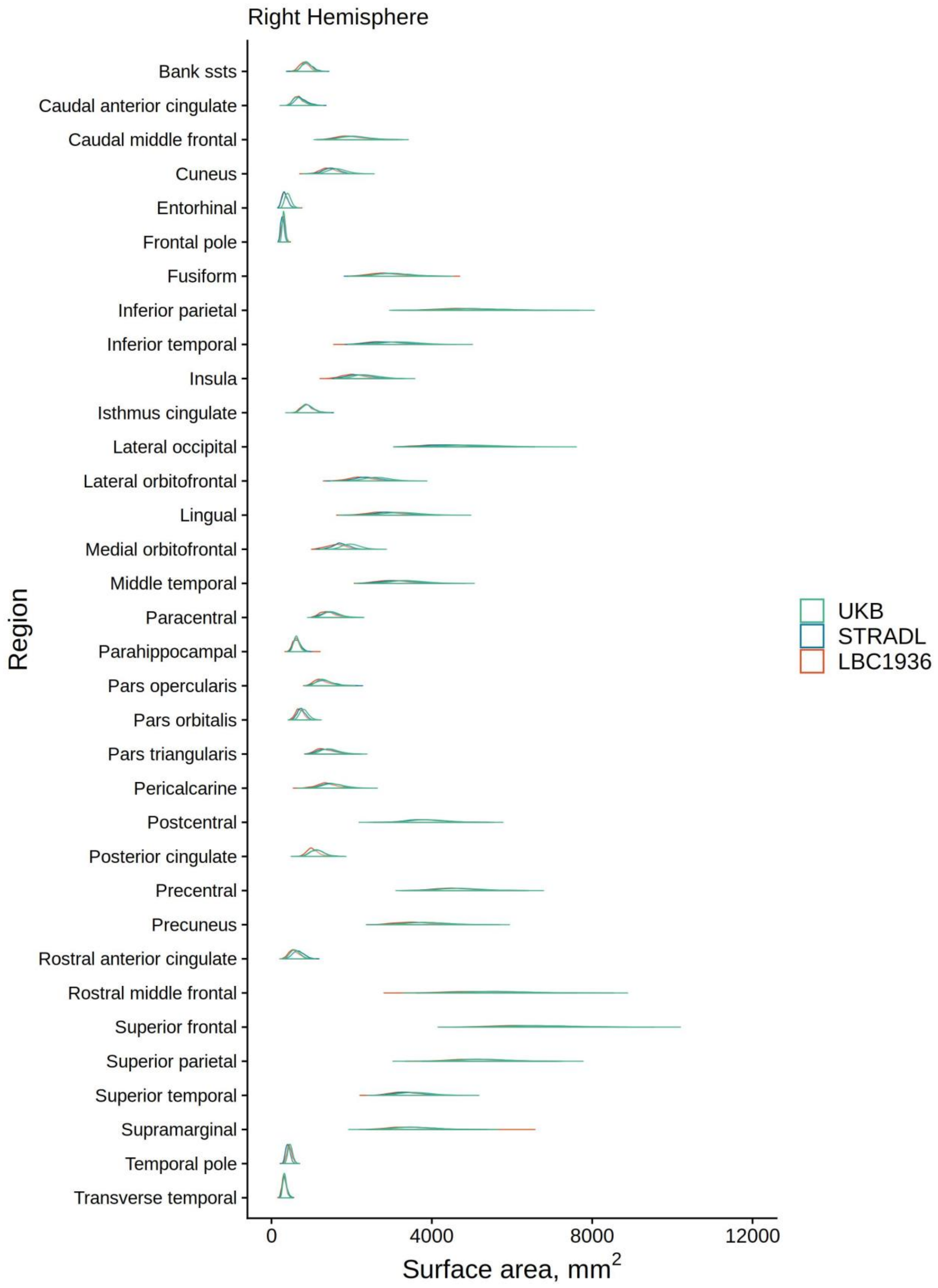

Figure S10 Regional density plots of right hemisphere surface area (mm2), for UKB, STRADL and LBC1936.

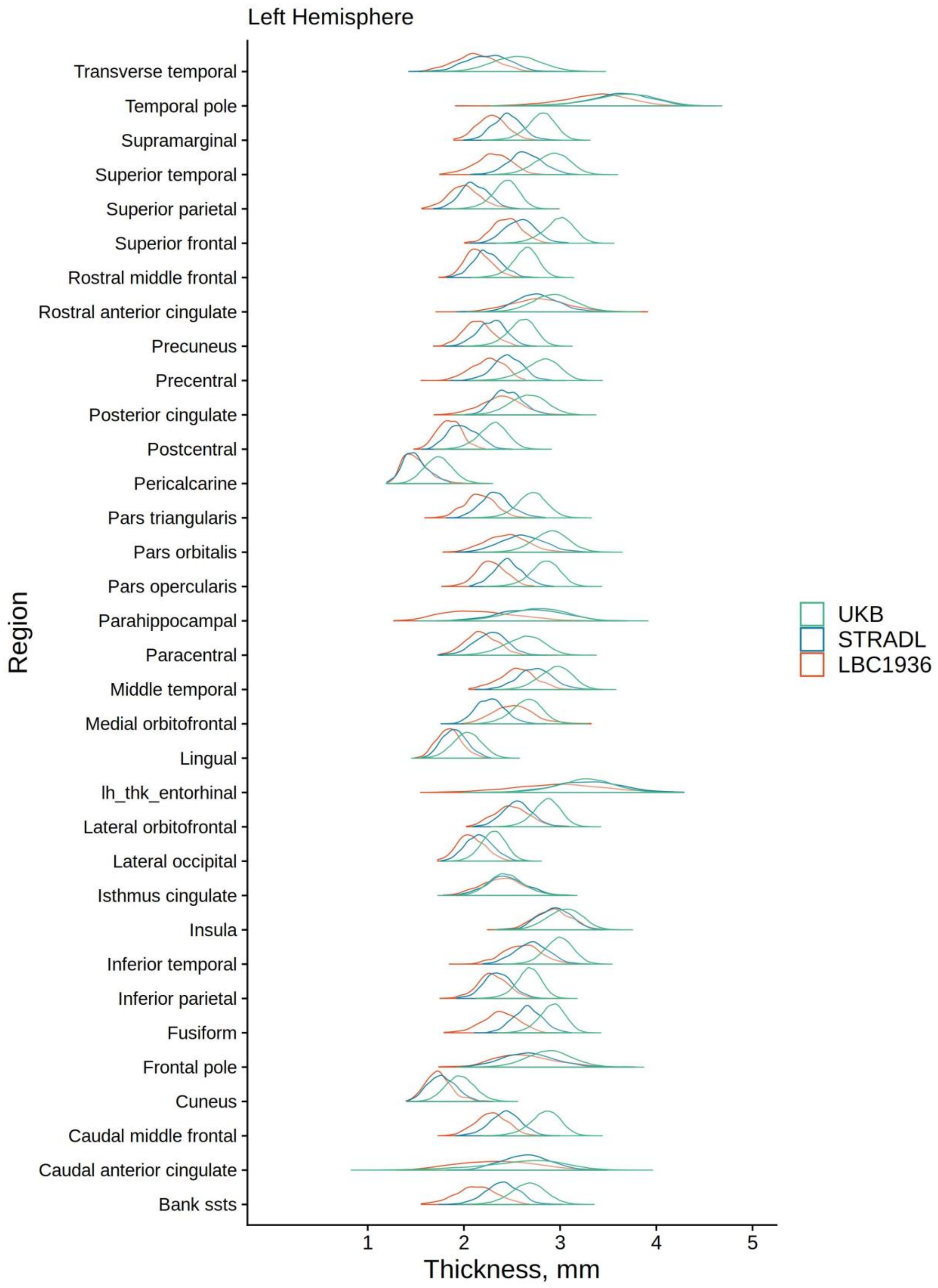

Figure S11 Regional density plots of left hemisphere thickness (mm), for UKB, STRADL and LBC1936.

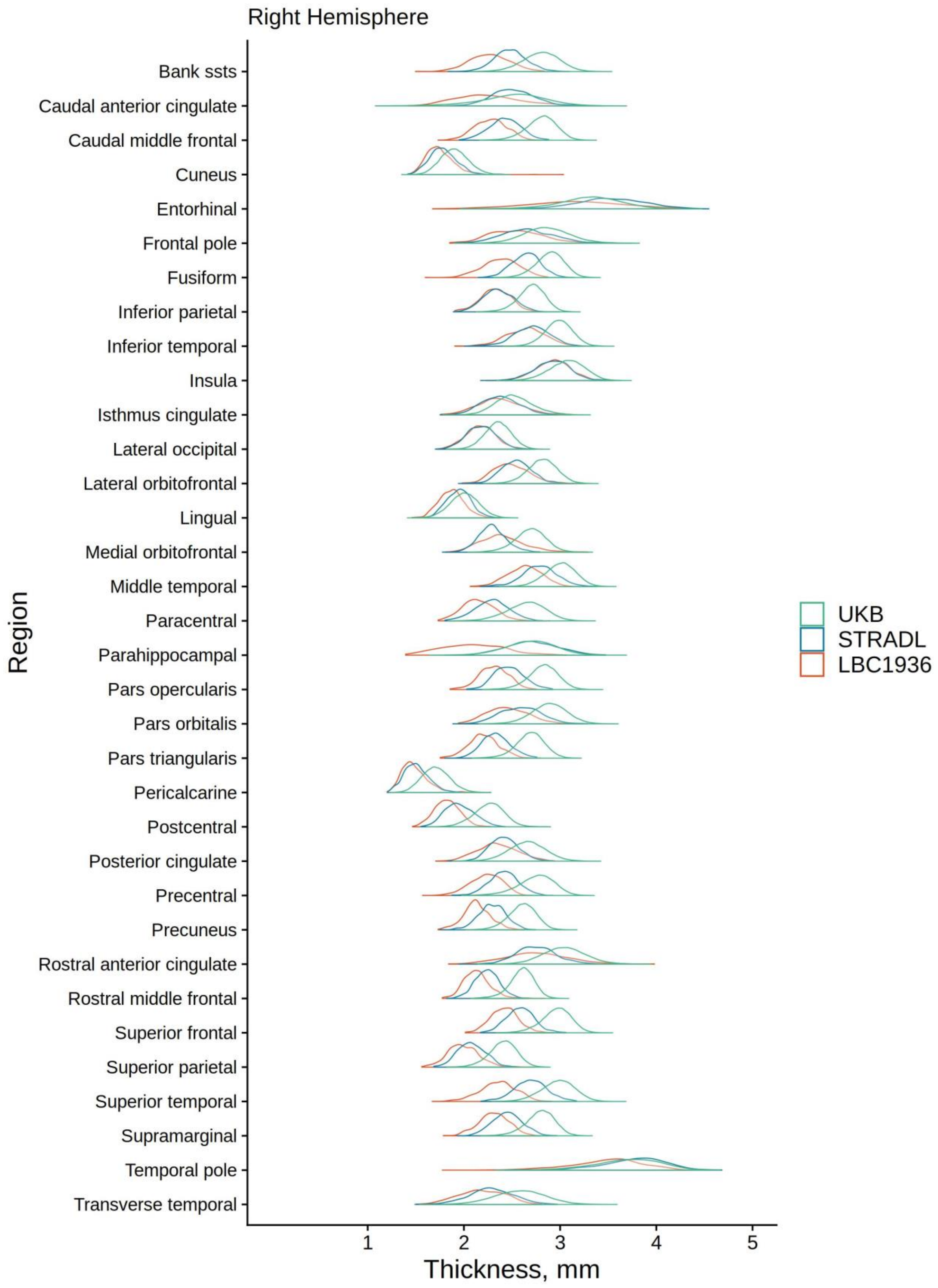

Figure S12 Regional density plots of right hemisphere thickness (mm), for UKB, STRADL and LBC1936.

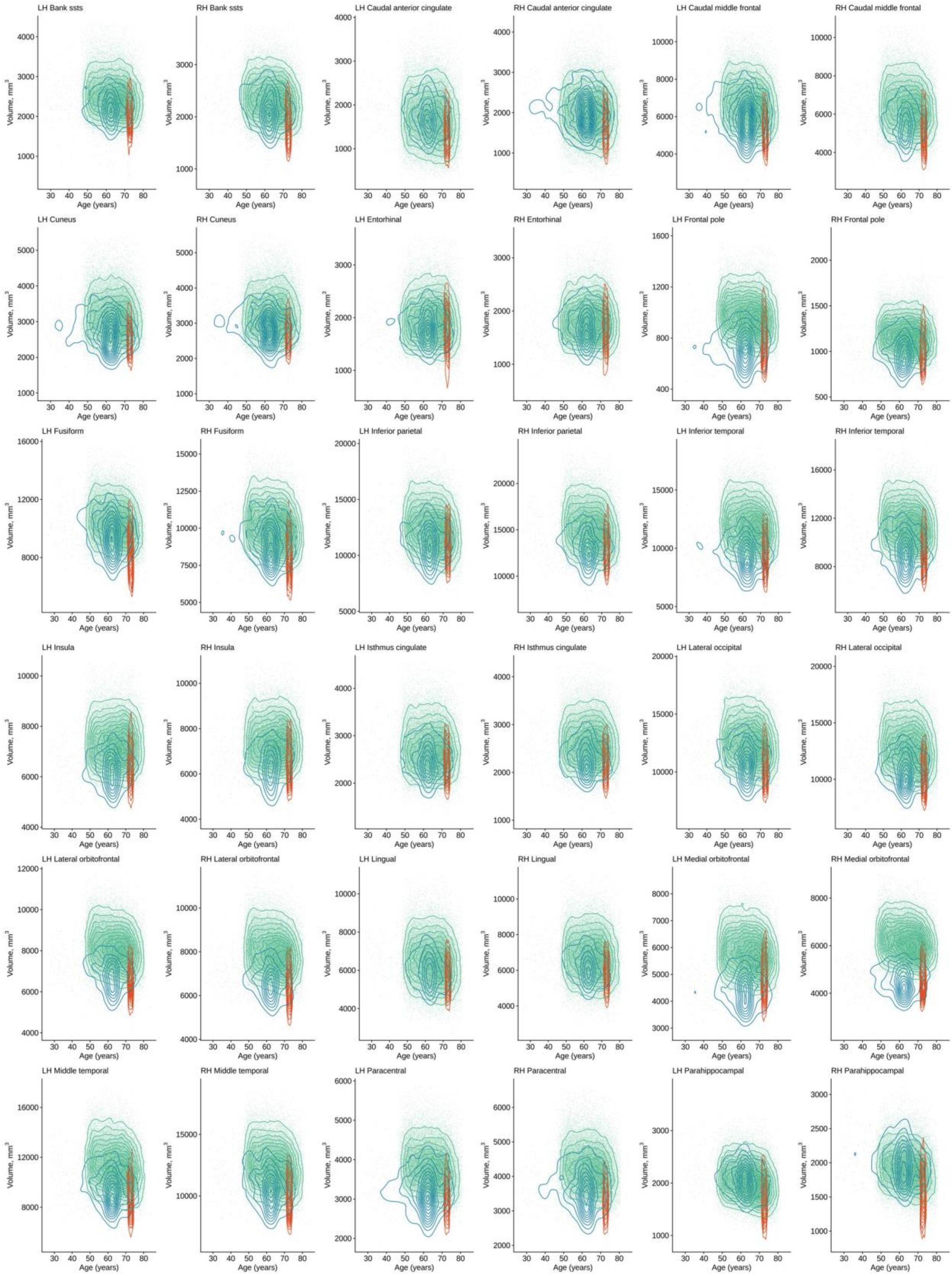
LH Bank ssts
RH Bank ssts
LH Caudal anterior cingulate
RH Caudal anterior cingulate
LH Caudal middle frontal
RH Caudal middle frontal
LH Cuneus
RH Cuneus
LH Entorhinal
RH Entorhinal
LH Frontal pole
RH Frontal pole
LH Fusiform
RH Fusiform
LH Inferior parietal
RH Inferior parietal
LH Inferior temporal
RH Inferior temporal
LH Insula
RH Insula
LH Isthmus cingulate
RH Isthmus cingulate
LH Lateral occipital
RH Lateral occipital
LH Lateral orbitofrontal
RH Lateral orbitofrontal
LH Lingual
RH Lingual
LH Medial orbitofrontal
RH Medial orbitofrontal
LH Middle temporal
RH Middle temporal
LH Paracentral
RH Paracentral
LH Parahippocampal
RH Parahippocampal
Volume, mm3
Age (years)

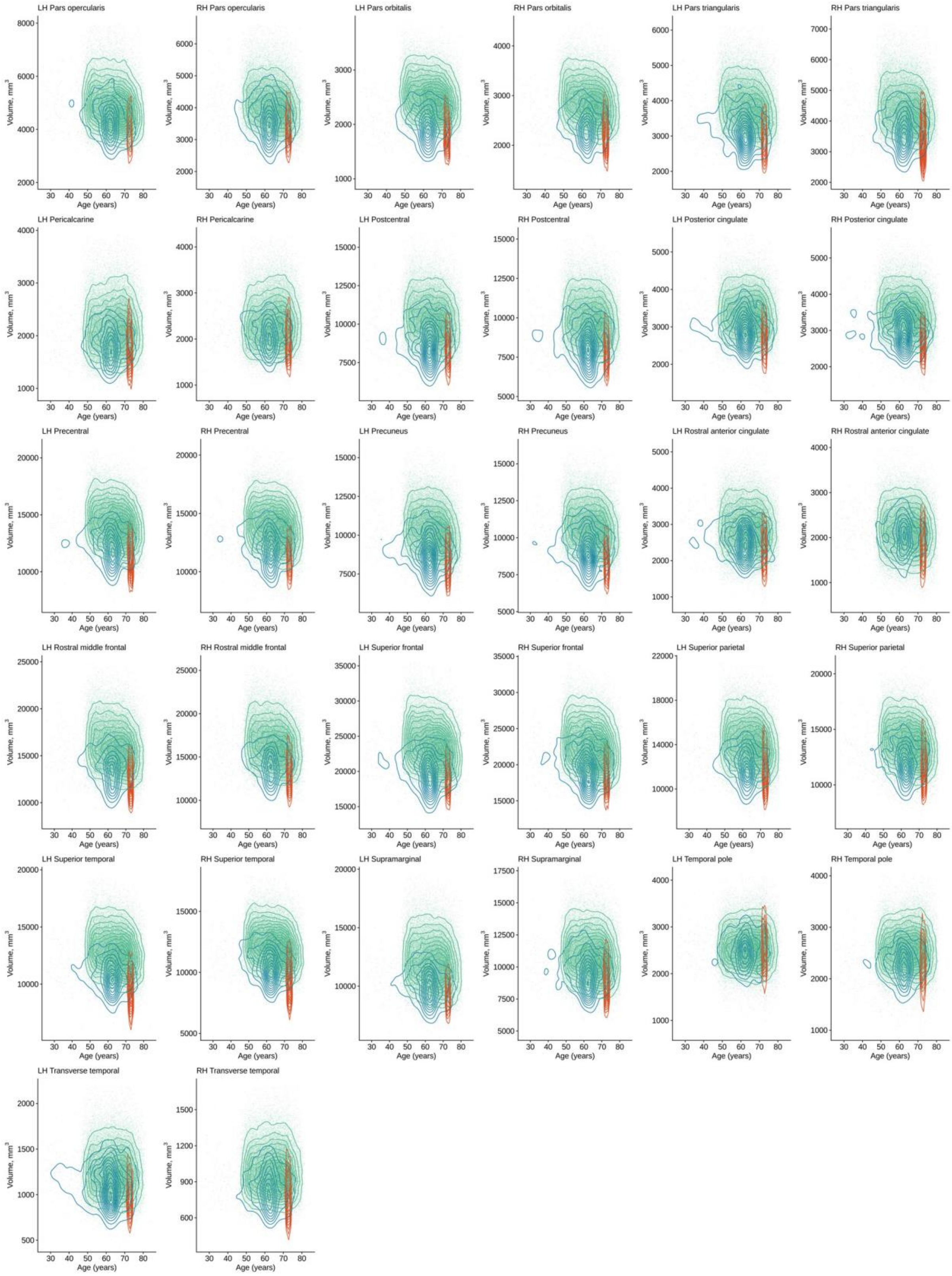

LH Pars opercularis
RH Pars opercularis
LH Pars orbitalis
RH Pars orbitalis
LH Pars triangularis
RH Pars triangularis
LH Pericalcarine
RH Pericalcarine
LH Postcentral
RH Postcentral
LH Posterior cingulate
RH Posterior cingulate
LH Precentral
RH Precentral
LH Precuneus
RH Precuneus
LH Rostral anterior cingulate
RH Rostral anterior cingulate
LH Rostral middle frontal
RH Rostral middle frontal
LH Superior frontal
RH Superior frontal
LH Superior parietal
RH Superior parietal
LH Superior temporal
RH Superior temporal
LH Supramarginal
RH Supramarginal
LH Temporal pole
RH Temporal pole
LH Transverse temporal
RH Transverse temporal
Volume, mm^3
Age (years)

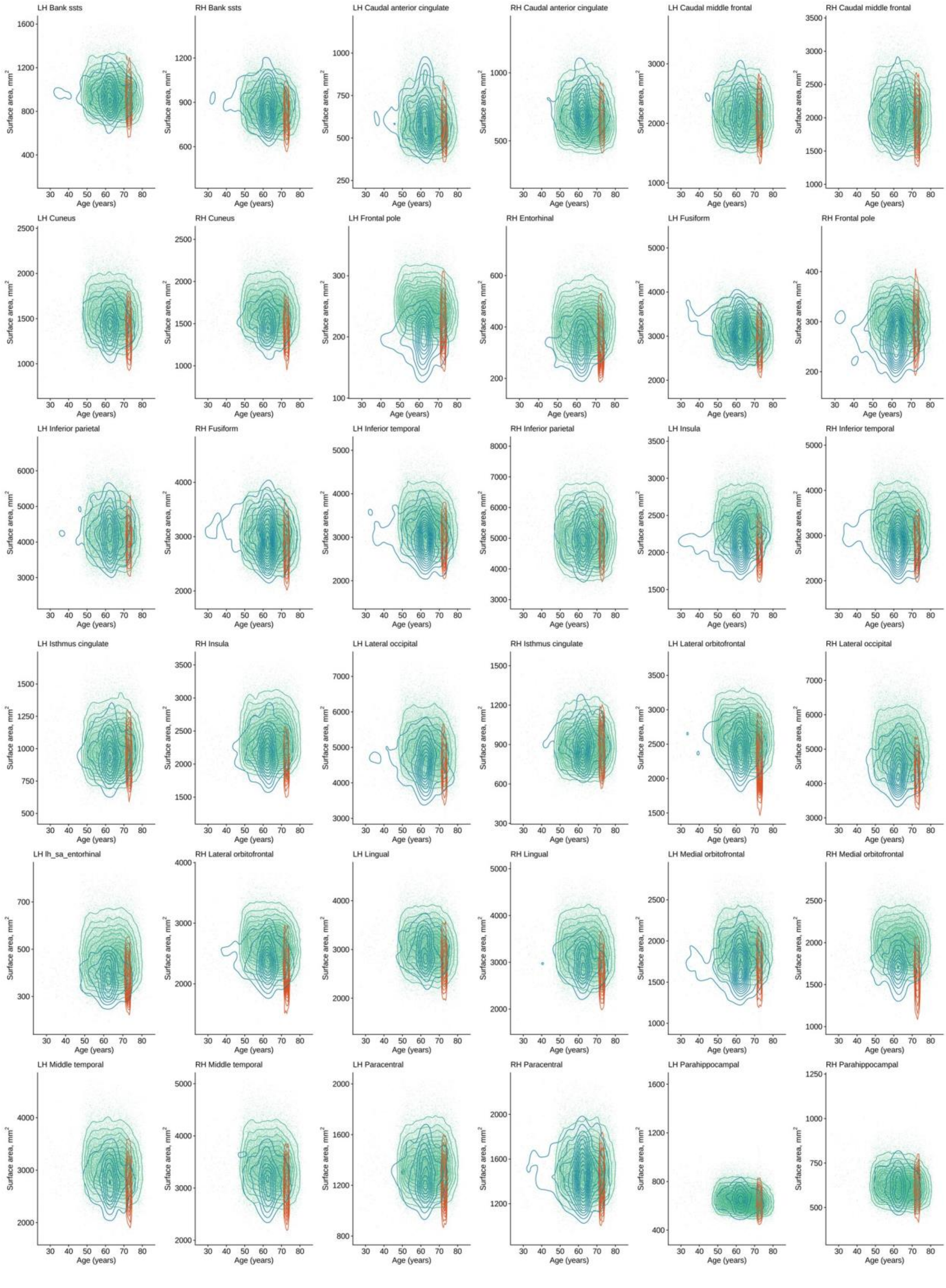

LH Bank ssts
RH Bank ssts
LH Caudal anterior cingulate
RH Caudal anterior cingulate
LH Caudal middle frontal
RH Caudal middle frontal
LH Cuneus
RH Cuneus
LH Frontal pole
RH Entorhinal
LH Fusiform
RH Frontal pole
LH Inferior parietal
RH Fusiform
LH Inferior temporal
RH Inferior parietal
LH Insula
RH Inferior temporal
LH Isthmus cingulate
RH Insula
LH Lateral occipital
RH Isthmus cingulate
LH Lateral orbitofrontal
RH Lateral occipital
LH lh_sa_entorhinal
RH Lateral orbitofrontal
LH Lingual
RH Lingual
LH Medial orbitofrontal
RH Medial orbitofrontal
LH Middle temporal
RH Middle temporal
LH Paracentral
RH Paracentral
LH Parahippocampal
RH Parahippocampal
Surface area, mm^2
Age (years)

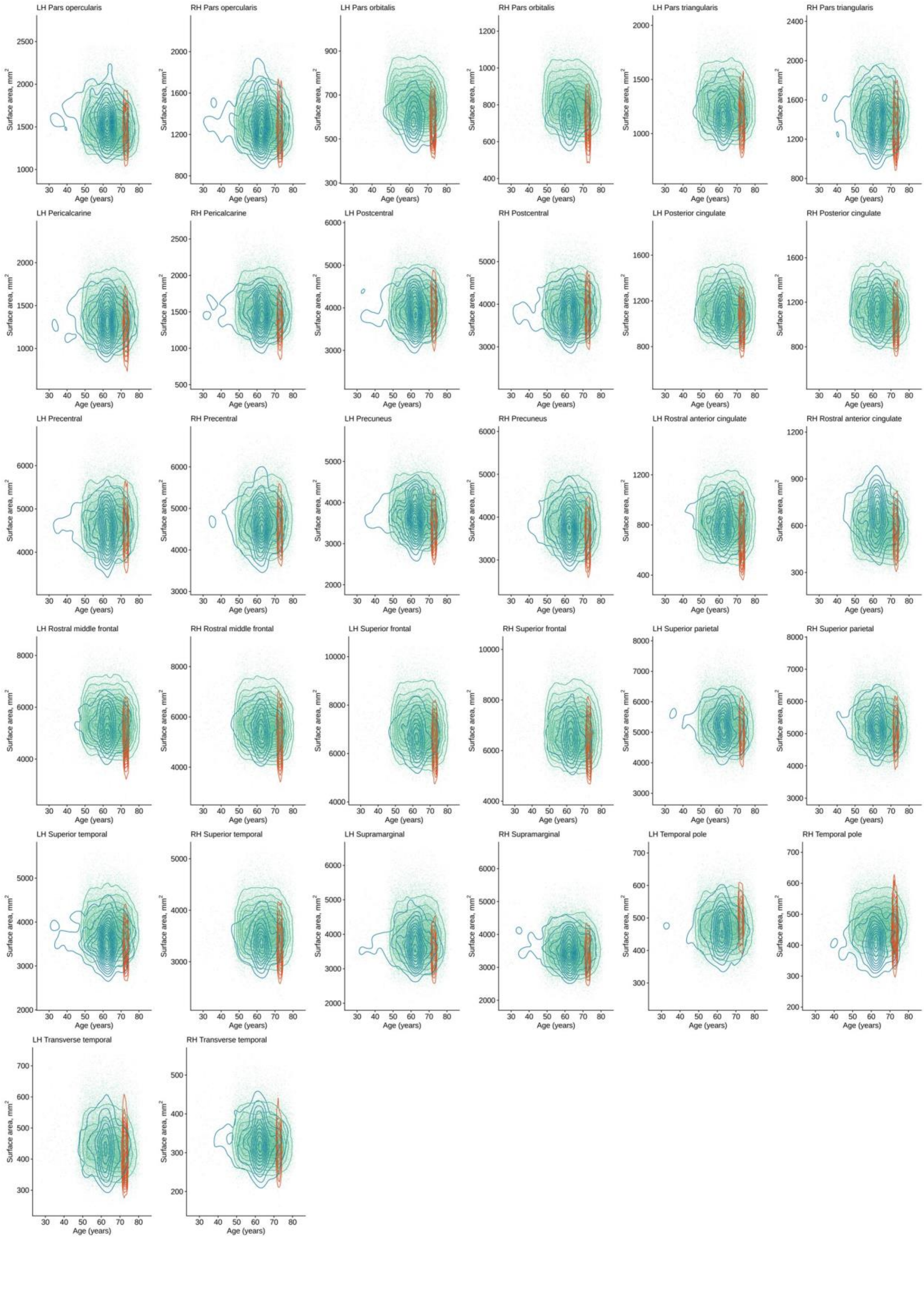
LH Pars opercularis
RH Pars opercularis
LH Pars orbitalis
RH Pars orbitalis
LH Pars triangularis
RH Pars triangularis
LH Pericalcarine
RH Pericalcarine
LH Postcentral
RH Postcentral
LH Posterior cingulate
RH Posterior cingulate
LH Precentral
RH Precentral
LH Precuneus
RH Precuneus
LH Rostral anterior cingulate
RH Rostral anterior cingulate
LH Rostral middle frontal
RH Rostral middle frontal
LH Superior frontal
RH Superior frontal
LH Superior parietal
RH Superior parietal
LH Superior temporal
RH Superior temporal
LH Supramarginal
RH Supramarginal
LH Temporal pole
RH Temporal pole
LH Transverse temporal
RH Transverse temporal
Surface area, mm^2
Age (years)

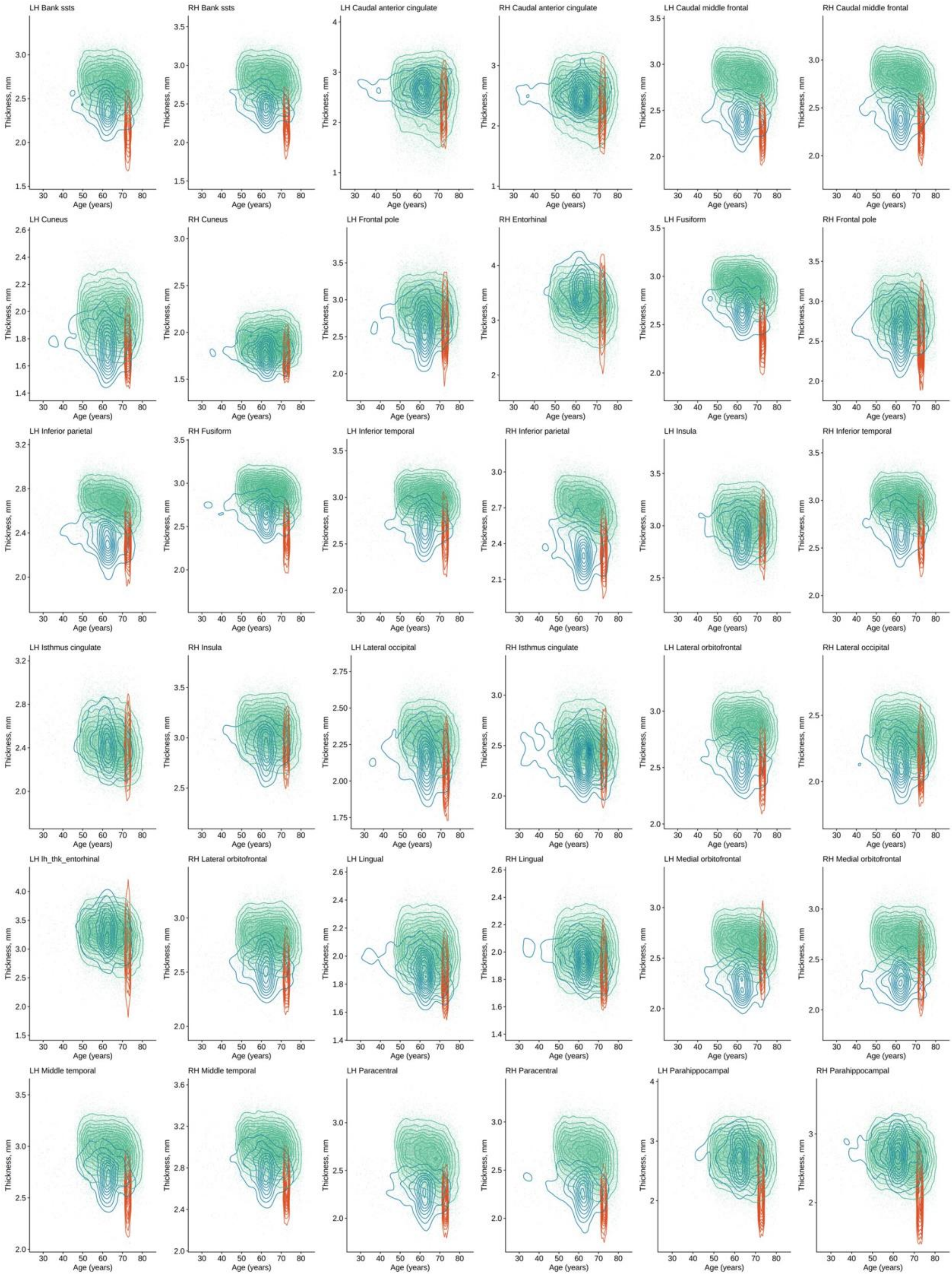
LH Bank ssts
RH Bank ssts
LH Caudal anterior cingulate
RH Caudal anterior cingulate
LH Caudal middle frontal
RH Caudal middle frontal
LH Cuneus
RH Cuneus
LH Frontal pole
RH Entorhinal
LH Fusiform
RH Frontal pole
LH Inferior parietal
RH Fusiform
LH Inferior temporal
RH Inferior parietal
LH Insula
RH Inferior temporal
LH Isthmus cingulate
RH Insula
LH Lateral occipital
RH Isthmus cingulate
LH Lateral orbitofrontal
RH Lateral occipital
LH lh_thk_entorhinal
RH Lateral orbitofrontal
LH Lingual
RH Lingual
LH Medial orbitofrontal
RH Medial orbitofrontal
LH Middle temporal
RH Middle temporal
LH Paracentral
RH Paracentral
LH Parahippocampal
RH Parahippocampal
Thickness, mm
Age (years)

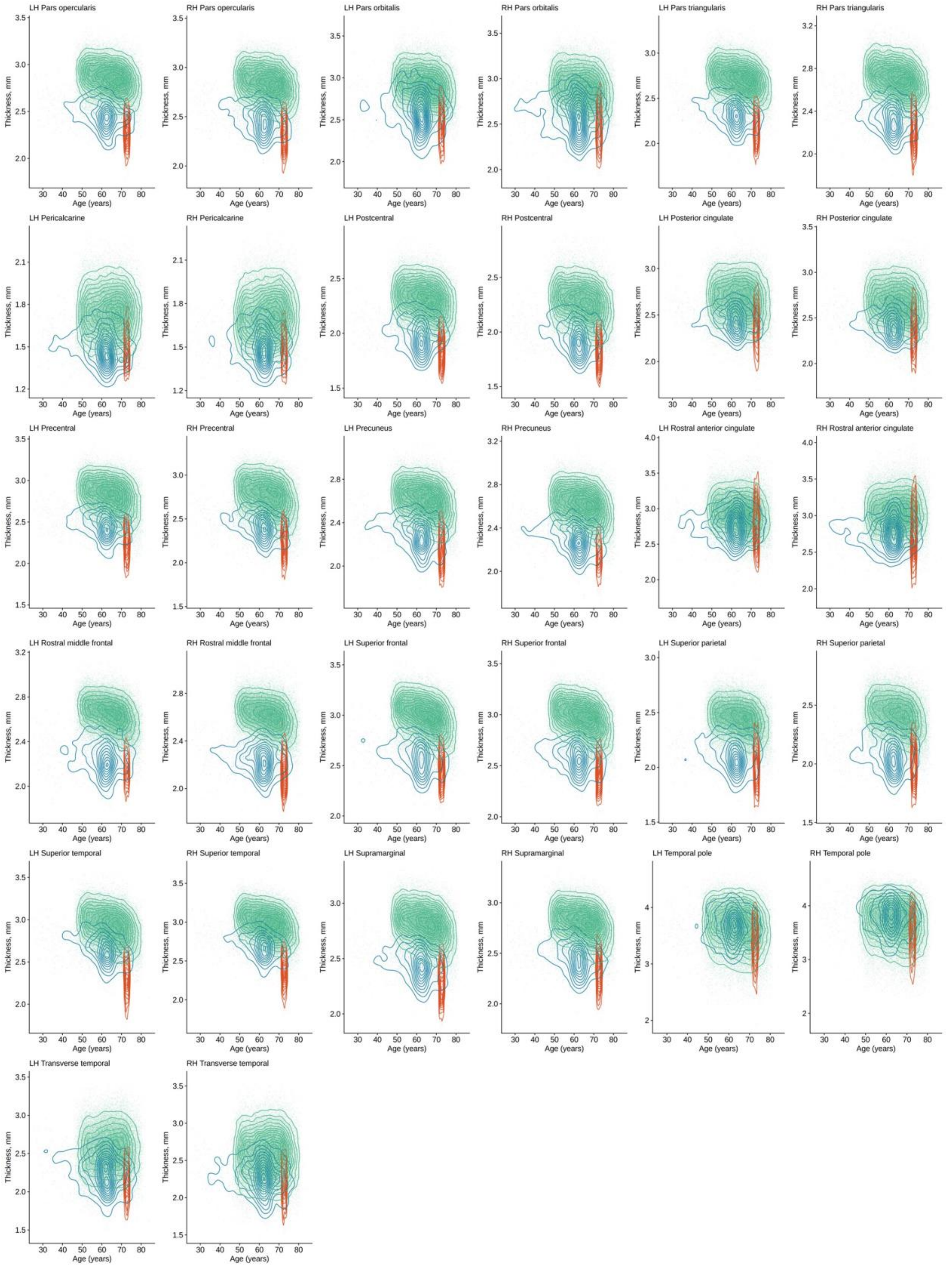

Figure S13 Regional volume (mm³), surface area (mm²), and thickness (mm) plotted by age for each cohort (UKB, STRADL and LBC).

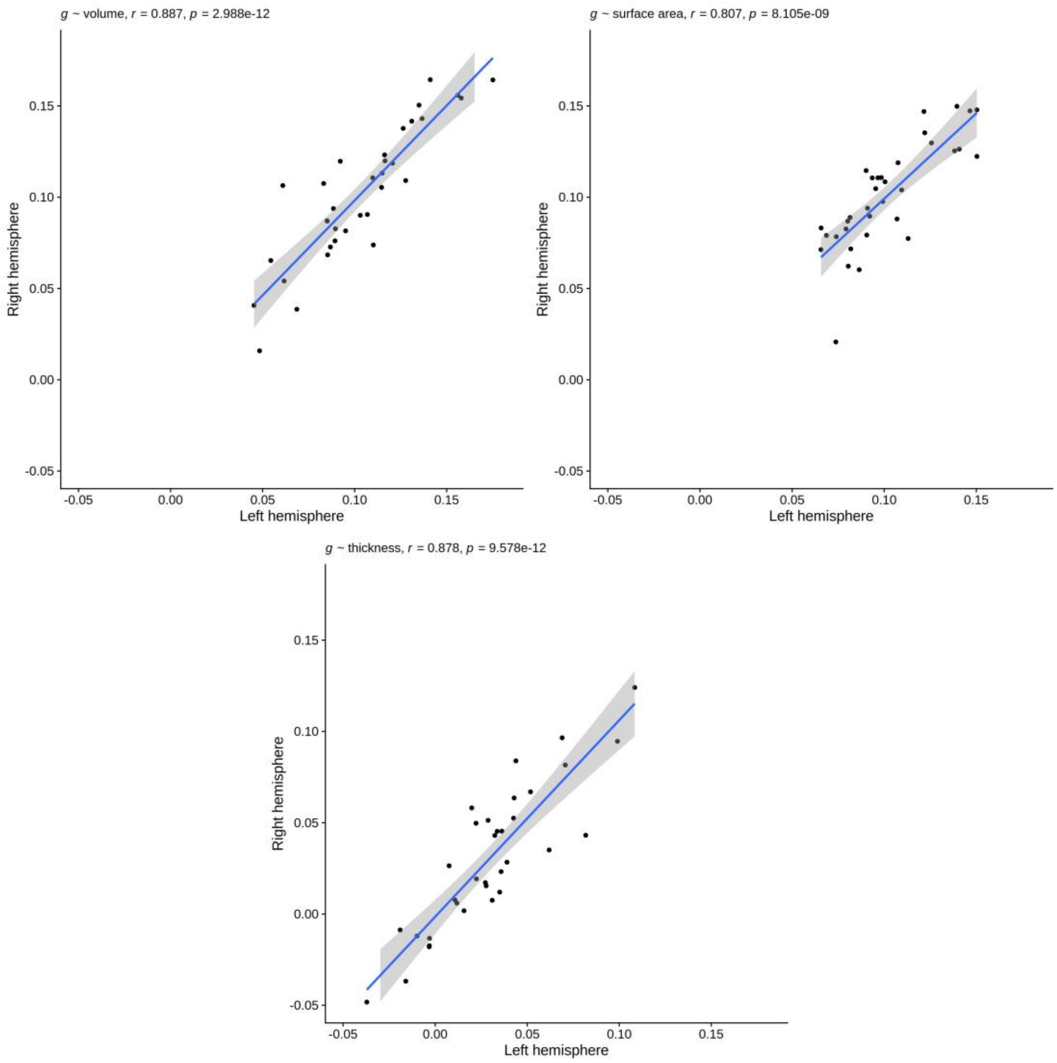

Figure S14 Inter-hemispheric correlations for meta-analysed g-associations with volume, surface area and thickness.

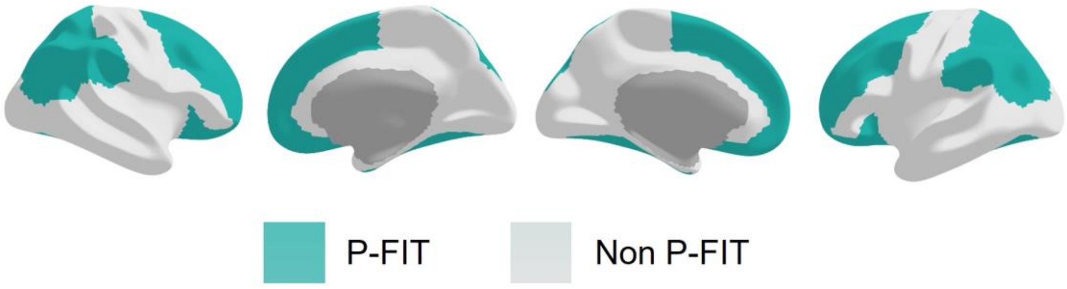

Figure S15 P-FIT and non-P-FIT regions mapped to the cortex (as in [2]).

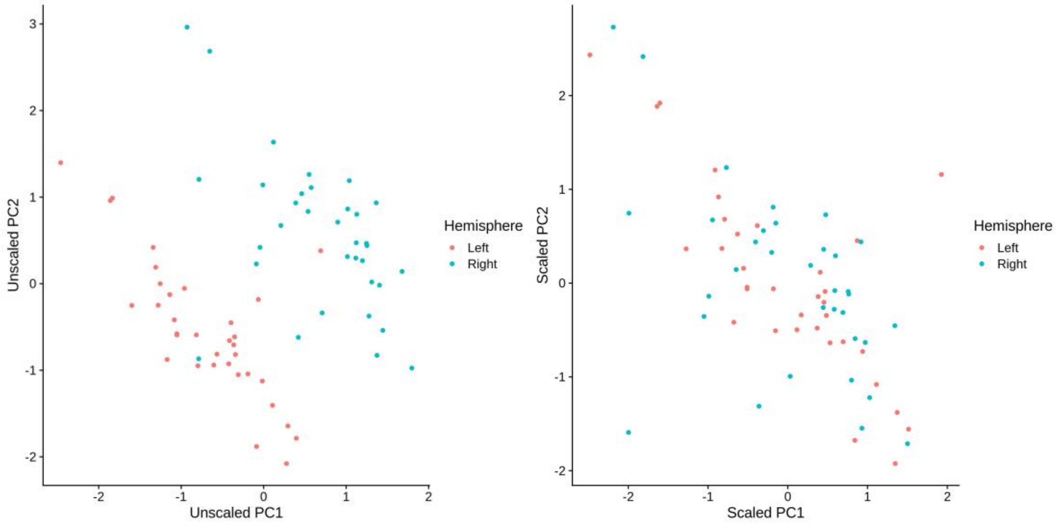

Figure S16 Rotated component scores (PC1 and PC2), unscaled (left) and scaled by hemisphere (right).

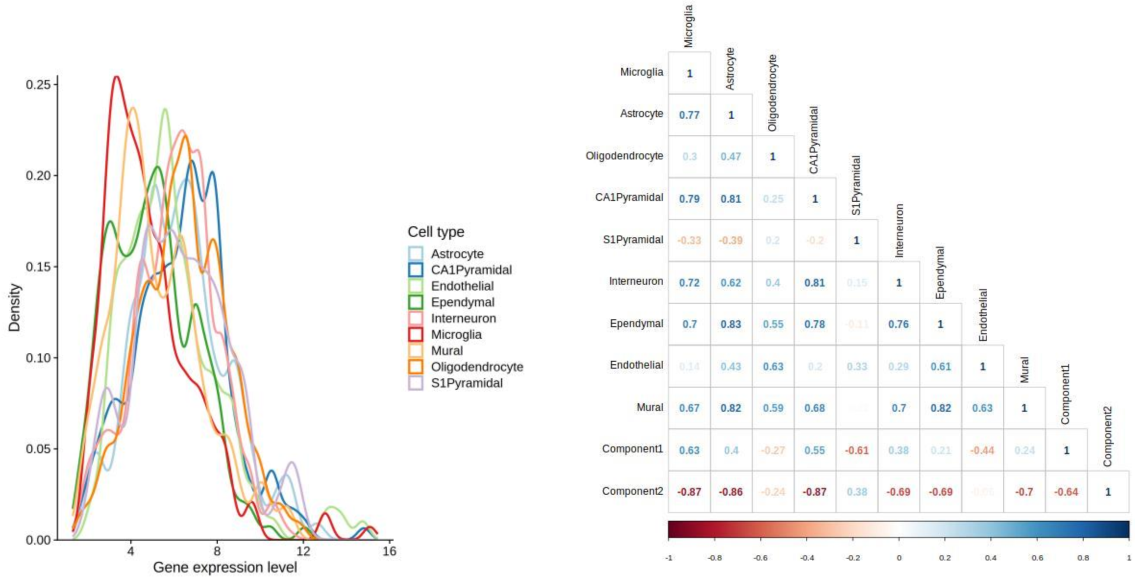

Figure S17 Raw expression values by the 9 cell types

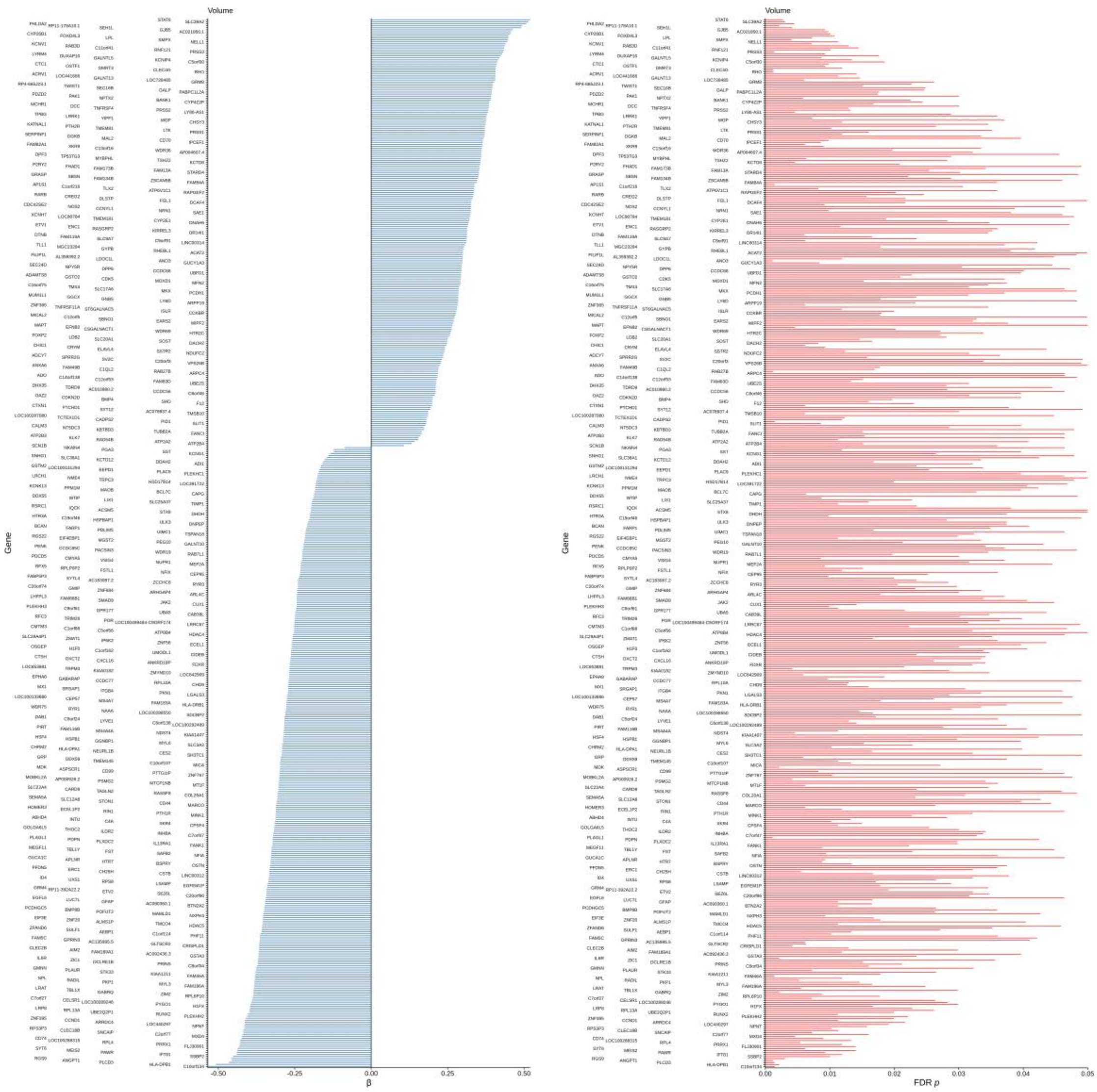

Figure S18 Regional gene-g associations that have FDR Q values < .05 for volume (N = 522).

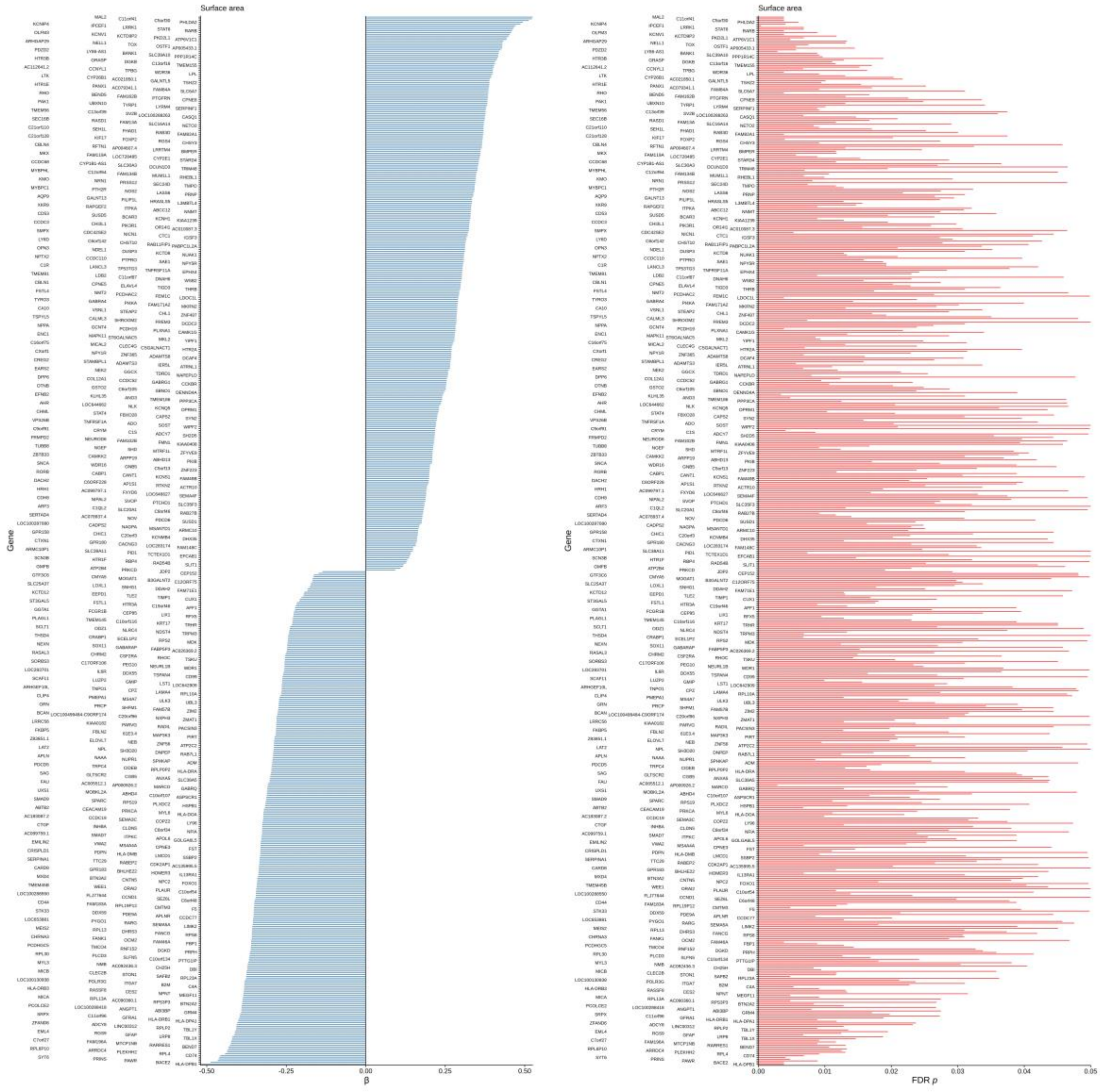

Figure S19 Regional gene-*g* associations that have FDR *Q* values < .05 for surface area (N = 609).

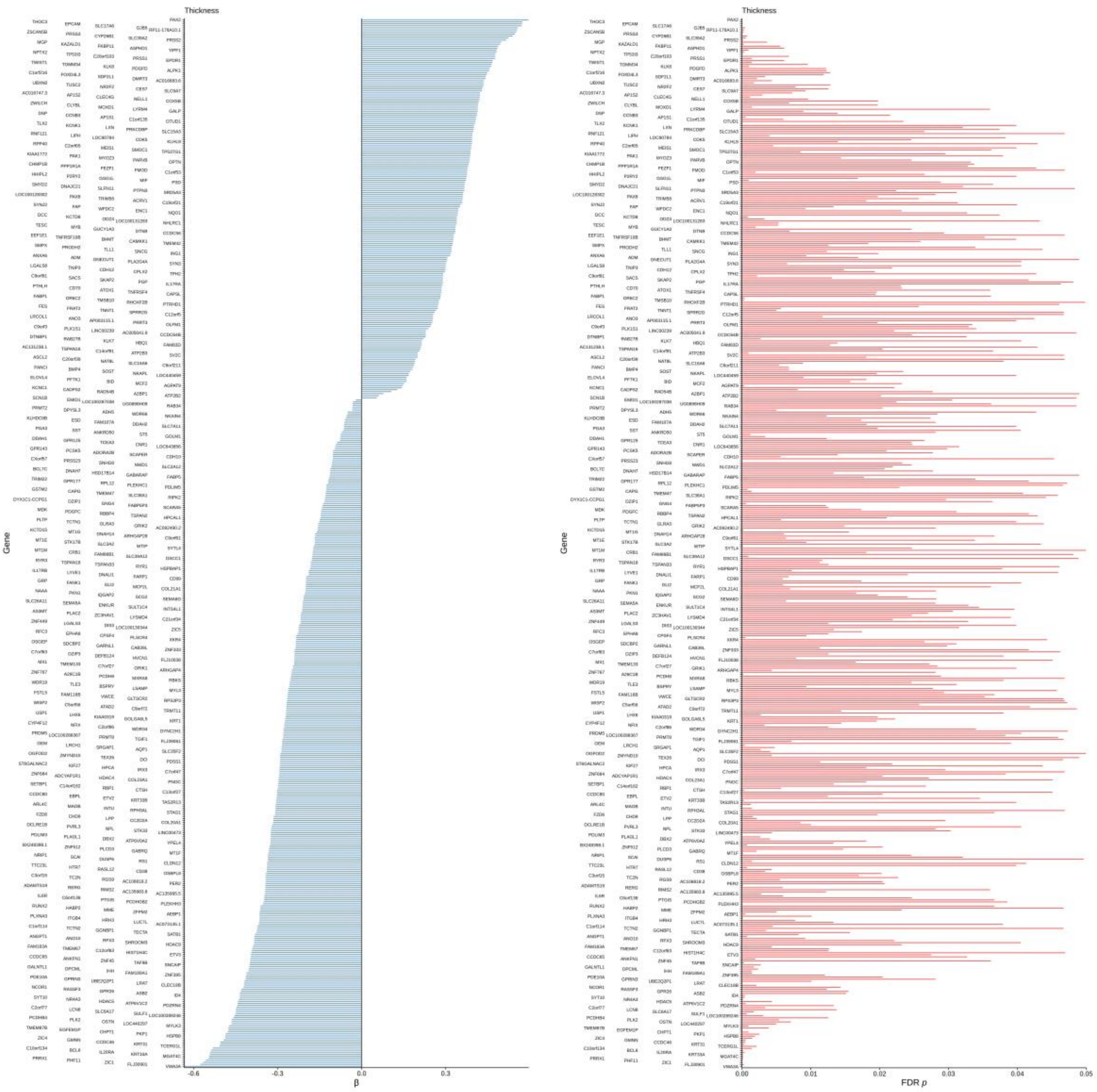

Figure S20 Regional gene-g associations that have FDR Q values < .05 for thickness ($N = 516$).

Table S1 Descriptive statistics of donor characteristics in the subsets of the three gene expression cohorts that are included in current analyses.

	Age (years)			Sex (*N*)		Donor *N*, *M (SD)*	
Dataset	M	SD	Range	Female	Male	Left hemisphere	Right hemisphere
Allen Atlas	42.50	13.38	24–57	1	5	5.71 (0.72)	2.00 (0.00)
BrainSpan	33.20	6.76	23–40	2	3	5 (0) – combined hemispheres	
Kang et al.	35.91	8.84	23–55	4	7	9.55 (1.04)	7.19 (0.60)

Table S2 Number of donors and samples for each of the 68 regions in the French and Paus (2015) expression matrix (ordered from smallest to largest number of samples in the right hemisphere region).

	Number of donors		Number of samples	
Region	Left hemisphere	Right hemisphere	Left hemisphere	Right hemisphere
Bank ssts	4	2	6	2
Frontal pole	3	2	9	2
Pars orbitalis	6	2	18	3
Entorhinal	6	2	18	4
Pars opercularis	5	2	13	4
Pericalcarine	5	2	8	4
Parstriangularis	5	2	7	5
Rostral anterior cingulate	6	2	18	5
Transverse temporal	6	2	14	5
Cuneus	6	2	30	6
Caudal anterior cingulate	6	2	28	7
Lateral occipital	6	2	45	8
Paracentral	6	2	28	8
Caudal middle frontal	6	2	15	9
Isthmus cingulate	6	2	24	9
Temporal pole	4	2	17	9
Inferior parietal	6	2	37	10
Precuneus	6	2	40	10
Insula	6	2	33	11
Lingual	6	2	45	11
Superior parietal	6	2	44	11
Lateral orbitofrontal	6	2	27	12
Parahippocampal	6	2	33	12
Posterior cingulate	6	2	37	13
Supramarginal	6	2	38	13
Medial orbitofrontal	6	2	54	21
Precentral	6	2	68	23
Fusiform	6	2	64	24
Rostral middle frontal	6	2	47	25
Superior temporal	6	2	80	25
Middle temporal	6	2	77	27
Postcentral	6	2	64	27
Superior frontal	6	2	100	29
Inferior temporal	6	2	83	34

Table S3 Descriptive statistics for loading distributions by cell types for both the two major components of cortical gene expression.

		Component 1			Component 2		
Cell type	N	Mean *(SD)*	Skewness	Kurtosis	Mean*(SD)*	Skewness	Kurtosis
Astrocyte	129	-0.04 (0.49)	0.375	1.933	0.31 (0.39)	-0.797	2.582
CA1 Pyramidal	204	-0.03 (0.50)	0.254	1.839	0.21 (0.46)	-0.364	2.057
Endothelial	127	0.16 (0.47)	-0.277	1.982	0.08 (0.45)	-0.016	1.835
Ependymal	191	0.09 (0.49)	0.061	1.812	0.21 (0.45)	-0.471	2.229
Interneuron	181	0.06 (0.51)	-0.005	1.644	0.14 (0.50)	-0.187	1.695
Microglia	185	-0.11 (0.46)	0.506	2.279	0.33 (0.41)	-0.845	2.846
Mural	60	0.05 (0.47)	0.278	1.938	0.17 (0.48)	-0.207	1.752
Oligodendrocyte	139	0.17 (0.48)	-0.228	1.850	0.18 (0.47)	-0.294	1.992
S1 Pyramidal	155	0.10 (0.49)	-0.253	1.920	-0.05 (0.47)	0.200	1.917
Unclassified	6864	0.14 (0.49)	-0.145	1.788	0.15 (0.46)	-0.145	1.788

Table S4 Dunn pairwise comparison results for loadings on Component 1 of cortical gene expression.

	Astrocyte		CA1Pyramidal		Endothelial		Ependymal		Interneuron		Microglia		Mural		Oligodendrocyte		S1 pyramidal	
Cell type	*z*	*p*	*z*	*p*	*z*	*p*	*z*	*p*	*z*	*p*	*z*	*p*	*z*	*p*	*z*	*p*	*z*	*p*
CA1Pyramidal	0.032	0.974																
Endothelial	**-3.193**	**0.048**	**-3.563**	**0.014**														
Ependymal	-2.318	0.614	-2.660	0.250	1.179	1.000												
Interneuron	-1.866	1.000	-2.141	0.903	1.590	1.000	0.474	1.000										
Microglia	1.148	1.000	1.261	1.000	**4.606**	**1.73e-04**	**3.837**	**0.005**	**3.316**	**0.032**								
Mural	-1.231	1.000	-1.335	1.000	1.320	1.000	0.485	1.000	0.152	1.000	-2.181	0.846						
Oligodendrocyte	**-3.534**	**0.015**	**-3.962**	**0.003**	-0.269	1.000	-1.506	1.000	-1.925	1.000	**-5.022**	***	-1.552	1.000				
S1Pyramidal	-2.437	0.459	-2.760	0.191	0.908	1.000	-0.243	1.000	-0.689	1.000	**-3.876**	**0.004**	-0.645	1.000	1.212	1.000		
Unclassified	**-4.051**	**0.002**	**-5.118**	**1.36E-05**	0.437	1.000	-1.307	1.000	-1.925	1.000	**-6.599**	**1.86E-09**	-1.293	1.000	0.841	1.000	-0.856	1.000

Table S5 Dunn pairwise comparison results for loadings on Component 2 of cortical gene expression.

	Astrocyte		CA1 Pyramidal		Endothelial		Ependymal		Interneuron		Microglia		Mural		Oligodendrocyte		S1 pyramidal	
Cell type	*z*	*p*	*z*	*p*	*z*	*p*	*z*	*p*	*z*	*p*	*z*	*p*	*z*	*p*	*z*	*p*	*z*	*p*
CA1 Pyramidal	1.839	1.000																
Endothelial	**3.950**	**0.003**	2.538	0.312														
Ependymal	1.929	1.000	0.129	0.897	-2.393	0.452												
Interneuron	3.105	0.063	1.478	1.000	-1.175	1.000	1.330	1.000										
Microglia	-0.499	1.000	-2.602	0.269	**-4.782**	*******	-2.686	0.217	**-3.970**	**0.003**								
Mural	1.842	1.000	0.551	1.000	-1.314	1.000	0.460	1.000	-0.470	1.000	2.323	0.525						
Oligodendrocyte	2.185	0.722	0.548	1.000	-1.846	1.000	0.424	1.000	-0.804	1.000	2.890	0.119	-0.134	1.000				
S1Pyramidal	**6.334**	**1.05E-08**	**5.143**	**1.14E-05**	2.181	0.700	**4.949**	**2.98E-05**	**3.628**	**9.71E-03**	**7.458**	**3.95E-12**	3.071	0.068	**4.175**	**0.001**		
Unclassified	**3.894**	**0.003**	1.959	1.000	-1.650	1.000	1.721	1.000	-0.156	1.000	**5.413**	**2.66E-06**	0.449	1.000	0.921	1.000	**-5.033**	**1.98E-05**

Table S6 The initial number of retained genes, and the number of genes that were matched to French and Paus' post-consistency check genes, alongside processing choices for each of the pipelines provided by Markello et al. (2021). Highlighted cells may help to explain some of the low factor congruence coefficients for PC2, compared to the French and Paus dataset.

Pipeline	Start *N* genes	*N* matched genes	reannotated	corrected_mni	tolerance (mm)	sample_norm	gene_norm	missing	probe_selection	donor_probes	region_agg	agg_metric	lr_mirror	ibf_threshold	norm_matched	norm_structures	sim_threshold
French & Paus (2015)	20739	8108	FALSE	FALSE	1	None	None	None	average	aggregate	donors	median	none	0	TRUE	FALSE	none
Anderson (2018)	20739	8108	FALSE	FALSE	0	None	center	None	corr_intensity	aggregate	donors	mean	none	0	TRUE	TRUE	none
Burt (2018)	20739	8108	FALSE	FALSE	2	zscore	zscore	interpolate	corr_variance	independent	donors	mean	none	0	TRUE	FALSE	5
Hawrylycz (2015)	20739	8108	FALSE	FALSE	0	None	zscore	cetroids	diff_stability	aggregate	donors	mean	none	0	TRUE	FALSE	none
Krienen (2016)	20739	8108	FALSE	FALSE	0	None	center	None	average	aggregate	donors	mean	mpme	0	TRUE	FALSE	none
Whitaker (2016)	20739	8108	FALSE	FALSE	0	None	zscore	centroids	average	aggregate	donors	mean	none	0	TRUE	FALSE	none
Romero-Garcia (2018)	20233	6800	TRUE	FALSE	0	None	zscore	interpolate	max_intensity	aggregate	samples	median	rightleft	0	TRUE	FALSE	none
Anderson (2020)	17383	7800	FALSE	FALSE	-4	zscore	zscore	None	max_intensity	aggregate	donors	mean	none	0.2	TRUE	FALSE	none
Liu (2020)	15631	6164	TRUE	FALSE	5	zscore	zscore		average	aggregate	donors	mean	none	0.5	FALSE	FALSE	none
Markello (2021)	15634	6166	TRUE	TRUE	2	srs	srs	None	diff_stability	aggregate	donors	mean	none	0.5	TRUE	FALSE	none

Table S7 Number of retained genes in each validation dataset after within-sample between-donor consistency measures, and the number that were matched with the 8235 consistent genes in the Allen dataset.

Data source	Start (*N*)	Consistent genes (*N*)	Matched with Allen 8235 genes (*N*)
BrainSpan	17604	4110	2250
Kang left hemisphere	17565	2030	1554
Kang right hemisphere	17565	2778	1784
Kang both hemispheres	17565	1048	908

Table S8 The initial number of retained genes, and the number of genes that were matched to French and Paus' post-consistency check gene for each pipeline following Markello et al.'s scripts([3]).

Pipeline	Start *N*	Matched *N*
Anderson (2018)	20739	8108
Burt (2018)	20739	8108
Hawrylycz (2015)	20739	8108
Krienen (2016)	20739	8108
Whitaker (2016)	20739	8108
Romero-Garcia (2018)	20233	6800
Anderson (2020)	17383	7800
Liu (2020)	15631	6164
Markello (2021)	15634	6166

Table S9 Regional component scores for the two major components of gene expression.

Hemisphere	Region	Component 1	Component 2
Left	Bank ssts	-0.382	0.613
	Caudal anterior cingulate	0.841	-1.677
	Caudal middle frontal	-0.180	-0.061
	Cuneus	-1.603	1.921
	Entorhinal	1.347	-1.925
	Frontal pole	1.926	1.159
	Fusiform	0.531	-0.637
	Inferior parietal	-0.630	0.523
	Inferior temporal	0.369	-0.480
	Insula	0.694	-0.626
	Isthmus cingulate	-0.510	-0.061
	Lateral occipital	-0.912	1.206
	Lateral orbitofrontal	0.468	-0.089
	Lingual	-1.638	1.885
	Medial orbitofrontal	0.938	-0.730
	Middle temporal	0.115	-0.497
	Paracentral	-0.554	0.158
	Parahippocampal	1.111	-1.081
	Pars opercularis	0.483	-0.346
	Pars orbitalis	-0.154	-0.508
	Pars triangularis	0.871	0.452
	Pericalcarine	-2.480	2.434
	Postcentral	-1.274	0.366
	Posterior cingulate	0.381	-0.144
	Precentral	-0.510	-0.044
	Precuneus	-0.792	0.681
	Rostral anterior cingulate	1.372	-1.379
	Rostral middle frontal	0.407	0.116
	Superior frontal	0.454	-0.205
	Superior parietal	-0.826	0.369
	Superior temporal	0.169	-0.340
	Supramarginal	-0.676	-0.417
	Temporal pole	1.514	-1.558
	Transverse temporal	-0.869	0.919
Right	Bank ssts	-1.996	-1.592
	Caudal anterior cingulate	0.799	-1.035
	Caudal middle frontal	0.449	0.360
	Cuneus	-1.815	2.416
	Entorhinal	1.501	-1.713
	Frontal pole	-0.361	-1.312
	Fusiform	0.444	-0.260
	Inferior parietal	-0.647	0.145
	Inferior temporal	0.758	-0.090
	Insula	0.972	-0.632
	Isthmus cingulate	-0.150	0.639
	Lateral occipital	-0.770	1.232
	Lateral orbitofrontal	0.691	-0.313
	Lingual	-1.992	0.746
	Medial orbitofrontal	0.844	-0.592
	Middle temporal	0.589	-0.080
	Paracentral	-0.990	-0.139
	Parahippocampal	1.342	-0.454
	Pars opercularis	0.030	-0.994
	Pars orbitalis	0.475	0.729
	Pars triangularis	0.918	0.440
	Pericalcarine	-2.188	2.730
	Postcentral	-0.944	0.674
	Posterior cingulate	0.764	-0.117
	Precentral	-0.404	0.439
	Precuneus	-0.306	0.560
	Rostral anterior cingulate	1.026	-1.221
	Rostral middle frontal	0.286	0.189
	Superior frontal	0.582	-0.279
	Superior parietal	-0.184	0.810
	Superior temporal	0.598	0.291
	Supramarginal	-1.050	-0.356
	Temporal pole	0.930	-1.547
	Transverse temporal	-0.202	0.328

Table S11 Brief descriptions of UKB cognitive tests and index codes.

Cognitive Test	Brief description	UKB field ID
Reaction time (s)	Time taken to respond in snap-type computer game	20023
Number span	Number of rounds completed (the maximum length of number string recalled)	4282
Fluid intelligence	Number of 13 verbal and numerical logic questions correct	20016
Trail making B (s) [4]	Time taken to complete an alphanumeric path-making test (trail B)	6350
Matrix pattern (log) [5]	Number of matrix pattern puzzles solved	6373
Tower task	Number of tower task puzzles solved	21003
Digit-symbol substitution [6]	Number of digit-symbol pairs matched	23324
Pairs matching [7]	Number of incorrect matches in a 6-pair classic pairs game	399
Prospective memory	Test of instruction recall after a delay	20018
Paired associates	Number of novel word pairs correctly recalled after a delay	20197
Picture vocabulary	Estimate of cognitive ability based on picture vocabulary task	26302

Table S12 Brief descriptions of STRADL cognitive tests and index codes.

Cognitive Test	Brief description	Code
Matrix reasoning ([5])	Number of puzzles correct	mrtotc
Verbal fluency ([8])	Number of words recalled beginning with C, F and L in 1 minute (1 minute per letter)	vftot
Mill Hill vocabulary ([9])	Number of word meanings explained correctly	mhv
Digit symbol substitution ([10])	Number of digit-symbol pairs matched	digsym
Logical memory ([10])	Story recall score (total from immediate and delayed tests)	mema + medela

Table S13 Brief descriptions of LBC1936 cognitive tests and index codes.

Cognitive test	Brief description	Code
Matrix reasoning ([10])	Number of puzzles correct	matreas_w2
Block design ([10])	Number of puzzles correct	blkdes_w2
Spatial span ([11])	Number of block sequences correct (forwards + backwards)	spantot_w2
National adult reading test (NART) ([12])	Number of words from the list pronounced correctly	nart_w2
Wechsler Test of Adult reading (WTAR) ([13])	Number of words from the list pronounced correctly	wtar_w2
Verbal fluency ([14])	Number of words recalled beginning with C, F and L i in 1 minute (1 minute per letter)	vftot_w2
Verbal paired associates ([11])	Number of novel word pairs recalled after a delay (total from immediate and delayed tests)	vpatotal_w2
Logical memory ([11])	Number of story details recalled (out of a total possible of 25) - total from immediate and delayed tests	lmtotal_w2
Digit span backwards ([10])	Max length of a string of numbers recalled in reverse	digback_w2
Symbol search	Number of symbols correctly detected during visual search	symsear_w2
Digit-symbol substitution ([15])	Number of digit-symbol pairs matched	digsym_w2
Inspection time ([16])	Number of correct responses at visual selection of longest line (two-alternative forced choice)	Ittotal_w2
Four-choice reaction time (s) ([17])	Time taken to press the indicated button (out of 4 buttons)	crtmean_w2

Table S14 UKB cognitive test summary statistics, and latent cognitive ability model estimates (for all paths to the latent factor, $p < .001$).

Cognitive Test	*N*	*M* (*SD*)	β	Residual variance
Reaction time (log)	35138	6.38 (0.17)	0.22 (0.006)	0.84
Numeric memory	25720	6.76 (1.27)	-0.46 (0.006)	0.76
Fluid intelligence	34709	6.60 (2.05)	-0.70 (0.004)	0.49
Trail making B (log)	24484	6.28 (0.36)	0.59 (0.005)	0.49
Matrix pattern	25135	7.95 (2.14)	-0.58 (0.005)	0.60
Tower task	24909	9.86 (3.23)	-0.49 (0.006)	0.71
Digit-symbol substitution	25134	18.87 (5.27)	-0.44 (0.005)	0.62
Pairs matching (log)	35365	1.35 (0.63)	0.24 (0.006)	0.92
Prospective memory	35350	0.84 (0.37)	0.31 (0.006)	0.88
Paired associates	25404	7.90 (02.64)	-0.44 (0.006)	0.75
Picture vocabulary	25077		-0.48 (0.006)	0.75

Table S15 STRADL cognitive test summary statistics and latent cognitive ability model estimates (for all paths to the latent factor, $p < .001$).

Cognitive Test	*N*	*M* (*SD*)	β (*SE*)	Residual variance
Matrix reasoning	1043	8.30 (2.39)	0.56 (0.029)	0.65
Verbal fluency	1043	43.10 (11.92)	0.53 (0.030)	0.71
Mill Hill vocabulary	1043	31.64 (4.07)	0.70 (0.028)	0.45
Digit symbol substitution	1043	68.77 (15.13)	0.37 (0.039)	0.64
Logical memory	1043	31.91 (7.23)	0.47 (0.030)	0.72

Table S16 LBC1936 cognitive test summary statistics, and cognitive ability model estimates (for all paths, besides the path between Verbal Memory and Cognitive ability which was fixed, all $p < .001$).

Cognitive test	*N*	*M (SD)*	β (*SE*)	Residual variance
Matrix reasoning	634	13.52 (4.93)	0.60 (0.03)	0.62
Block design	634	34.38 (10.01)	0.60 (0.03)	0.60
Spatial span	634	14.79 (2,72)	0.45 (0.04)	0.77
NART	634	34.66 (8.10)	0.63 (0.03)	0.57
WTAR	634	41.27 (6.94)	0.65 (0.03)	0.56
Phonemic verbal fluency	635	43.55 (12.78)	0.47 (0.04)	0.77
Verbal paired associates	623	27.57 (9.48)	0.51 (0.04)	0.70
Logical memory	635	75.03 (17.84)	0.52 (0.04)	0.71
Digit span backwards	636	7.88 (2.31)	0.55 (0.04)	0.69
Symbol search	634	24.88 (6.05)	0.58 (0.03)	0.64
Digit-symbol substitution	634	56.68 (11.79)	0.61 (0.03)	0.57
Inspection time	634	111.78 (10.95)	0.38 (0.04)	0.84
Four-choice reaction time (s)	635	0.64 (0.08)	0.39 (0.04)	0.82

*Table S17 W*ithin-domain residual variances for LBC1936 general cognitive ability model.

Cognitive test	β (*SE*)
Matrix reasoning ~~ block design	0.25 (0.05)
Matrix reasoning ~~ spatial span	0.09 (0.05)
Block design ~~ spatial span	0.20 (0.05)
NART ~~ WTAR	0.83 (0.02)
NART~~ verbal fluency	0.16 (0.05)
WTAR ~~ verbal fluency	0.16 (0.05)
Verbal paired associates ~~ logical memory	0.35 (0.04)
Verbal paired associates ~~ digit span backward	-0.04 (0.05)
Logical memory ~~ digit span backward	0.01 (0.05)
Symbol search ~~ digit symbol	0.40 (0.04)
Symbol search ~~ inspection time	0.17 (0.04)
Symbol search ~~ choice reaction time	0.31 (0.04)
Digit symbol ~~ inspection time	0.22 (0.04)
Digit symbol ~~ choice reaction time	0.36 (0.04)
Inspection time ~~ choice reaction time	0.25 (0.04)

Table S18 Model fits for the latent cognitive ability models*.*

Cohort	X^2	*df*	CFI	TLI	RMSEA	SRMR
UKB	3309	44	0.949	0.911	0.044	0.025
STRADL	1170	20	0.972	0.887	0.079	0.019
LBC1936	168	35	0.966	0.930	0.061	0.037

Table S19 Meta-analysis results for regional *g*-**volume** associations.

Volume	Left hemisphere								Right hemisphere							
Region	B	*SE*	*p*	FDR Q	Cochrane's *Q*	*p* (*Q*)	I^2	H^2	B	*SE*	*p*	FDR Q	Cochrane's *Q*	*p* (*Q*)	I^2	H^2
Bank ssts	0.110	0.031	0.000	0.001	7.785	0.020	0.002	5.082	0.111	0.024	0.000	0.000	5.993	0.050	0.001	3.272
Caudal anterior cingulate	0.045	0.005	0.000	0.000	0.607	0.738	0.000	1.000	0.041	0.005	0.000	0.000	0.533	0.766	0.000	1.000
Caudal middle frontal	0.107	0.025	0.000	0.000	5.673	0.059	0.001	3.425	0.091	0.009	0.000	0.000	2.101	0.350	0.000	1.125
Cuneus	0.062	0.005	0.000	0.000	0.415	0.812	0.000	1.000	0.054	0.033	0.096	0.098	7.231	0.027	0.003	5.842
Entorhinal	0.103	0.026	0.000	0.000	8.610	0.014	0.001	3.807	0.090	0.026	0.000	0.001	7.858	0.020	0.001	3.720
Frontal pole	0.048	0.005	0.000	0.000	2.338	0.311	0.000	1.002	0.016	0.015	0.301	0.301	3.014	0.222	0.000	1.569
Fusiform	0.158	0.039	0.000	0.000	17.520	0.000	0.004	9.872	0.154	0.037	0.000	0.000	18.194	0.000	0.003	8.804
Inferior parietal	0.141	0.031	0.000	0.000	11.869	0.003	0.002	5.418	0.164	0.041	0.000	0.000	22.780	0.000	0.004	11.305
Inferior temporal	0.137	0.049	0.005	0.005	17.043	0.000	0.006	14.800	0.143	0.053	0.007	0.007	20.177	0.000	0.008	18.648
Insula	0.116	0.004	0.000	< .001	1.015	0.602	< .001	1.000	0.123	0.027	< .001	< .001	8.333	0.016	0.002	4.978
Isthmus cingulate	0.085	0.012	< .001	< .001	2.575	0.276	< .001	1.398	0.068	0.005	< .001	< .001	0.454	0.797	< .001	1.000
Lateral occipital	0.085	0.004	< .001	< .001	1.009	0.604	< .001	1.000	0.087	0.010	< .001	< .001	2.808	0.246	< .001	1.221
Lateral orbitofrontal	0.128	0.017	< .001	< .001	4.094	0.129	< .001	2.064	0.109	0.005	< .001	< .001	2.752	0.253	< .001	1.004
Lingual	0.092	0.034	0.007	0.008	9.675	0.008	0.003	6.629	0.120	0.044	0.006	0.006	20.018	< .001	0.005	11.091
Medial orbitofrontal	0.089	0.005	< .001	< .001	0.110	0.947	< .001	1.000	0.094	0.004	< .001	< .001	0.954	0.621	< .001	1.000
Middle temporal	0.156	0.045	0.001	0.001	17.181	< .001	0.006	13.446	0.156	0.034	< .001	< .001	13.277	0.001	0.003	7.964
Paracentral	0.090	0.015	< .001	< .001	2.744	0.254	< .001	1.569	0.083	0.005	< .001	< .001	0.717	0.699	< .001	1.000
Parahippocampal	0.061	0.010	< .001	< .001	1.928	0.381	< .001	1.172	0.106	0.036	0.003	0.004	14.066	0.001	0.003	7.173
Pars opercularis	0.110	0.025	< .001	< .001	6.645	0.036	0.001	3.649	0.074	0.005	< .001	< .001	1.000	0.607	< .001	1.000
Pars orbitalis	0.089	0.005	< .001	< .001	0.255	0.880	< .001	1.000	0.076	0.018	< .001	< .001	4.025	0.134	0.001	2.013
Pars triangularis	0.087	0.005	< .001	< .001	3.070	0.215	< .001	1.015	0.073	0.005	< .001	< .001	0.571	0.752	< .001	1.000
Pericalcarine	0.054	0.005	< .001	< .001	1.276	0.528	< .001	1.000	0.065	0.017	< .001	< .001	3.249	0.197	< .001	1.761
Postcentral	0.115	0.027	< .001	< .001	6.100	0.047	0.002	4.297	0.113	0.020	< .001	< .001	5.208	0.074	0.001	2.616
Posterior cingulate	0.085	0.005	< .001	< .001	1.359	0.507	< .001	1.000	0.087	0.005	< .001	< .001	4.095	0.129	< .001	1.000
Precentral	0.135	0.022	< .001	< .001	5.651	0.059	0.001	3.012	0.150	0.032	< .001	< .001	11.780	0.003	0.003	6.422
Precuneus	0.131	0.025	< .001	< .001	8.113	0.017	0.001	4.113	0.142	0.029	< .001	< .001	11.525	0.003	0.002	5.587
Rostral anterior cingulate	0.095	0.017	< .001	< .001	3.741	0.154	< .001	1.816	0.082	0.016	< .001	< .001	3.020	0.221	< .001	1.681
Rostral middle frontal	0.121	0.017	< .001	< .001	4.228	0.121	< .001	2.110	0.119	0.018	< .001	< .001	4.610	0.100	0.001	2.289
Superior frontal	0.126	0.019	< .001	< .001	5.294	0.071	0.001	2.720	0.138	0.026	< .001	< .001	10.847	0.004	0.001	4.487
Superior parietal	0.116	0.028	< .001	< .001	7.174	0.028	0.002	4.684	0.120	0.032	< .001	< .001	9.309	0.010	0.002	6.162
Superior temporal	0.175	0.033	< .001	< .001	17.090	< .001	0.003	7.183	0.164	0.033	< .001	< .001	11.702	0.003	0.003	6.736
Supramarginal	0.115	0.018	< .001	< .001	4.279	0.118	0.001	2.150	0.105	0.023	< .001	< .001	5.156	0.076	0.001	3.283
Temporal pole	0.069	0.021	0.001	0.001	5.042	0.080	0.001	2.424	0.039	0.005	< .001	< .001	0.133	0.936	< .001	1.000
Transverse temporal	0.083	0.005	< .001	< .001	0.705	0.703	< .001	1.000	0.108	0.022	< .001	< .001	4.992	0.082	0.001	2.531

Table S20 Meta-analysis results for regional *g*-**surface area** associations.

Volume	Left hemisphere								Right hemisphere							
Region	B	*SE*	*p*	FDR Q	Cochrane's *Q*	*p* (*Q*)	I^2	H^2	B	*SE*	*p*	FDR Q	Cochrane's *Q*	*p* (*Q*)	I^2	H^2
Bank ssts	0.090	0.015	< .001	< .001	3.525	0.172	< .001	1.534	0.115	0.021	< .001	< .001	5.052	0.080	0.001	2.530
Caudal anterior cingulate	0.069	0.006	< .001	< .001	1.356	0.508	< .001	1.015	0.079	0.013	< .001	< .001	2.704	0.259	< .001	1.380
Caudal middle frontal	0.081	0.005	< .001	< .001	1.097	0.578	< .001	1.000	0.089	0.005	< .001	< .001	1.354	0.508	< .001	1.000
Cuneus	0.074	0.011	< .001	< .001	2.329	0.312	< .001	1.244	0.078	0.005	< .001	< .001	3.242	0.198	< .001	1.003
Entorhinal	0.080	0.016	< .001	< .001	3.189	0.203	< .001	1.686	0.062	0.005	< .001	< .001	0.162	0.922	< .001	1.000
Frontal pole	0.086	0.005	< .001	< .001	3.103	0.212	< .001	1.005	0.060	0.015	< .001	< .001	3.045	0.218	< .001	1.541
Fusiform	0.141	0.032	< .001	< .001	9.836	0.007	0.002	6.085	0.126	0.021	< .001	< .001	5.943	0.051	0.001	2.831
Inferior parietal	0.139	0.028	< .001	< .001	10.734	0.005	0.002	4.420	0.150	0.031	< .001	< .001	15.616	< .001	0.002	6.053
Inferior temporal	0.147	0.042	< .001	0.001	14.603	0.001	0.005	10.963	0.147	0.039	< .001	< .001	13.368	0.001	0.004	9.340
Insula	0.100	0.004	< .001	< .001	1.193	0.551	< .001	1.000	0.108	0.015	< .001	< .001	3.704	0.157	< .001	1.727
Isthmus cingulate	0.082	0.004	< .001	< .001	0.932	0.627	< .001	1.000	0.072	0.005	< .001	< .001	0.500	0.779	< .001	1.000
Lateral occipital	0.092	0.004	< .001	< .001	1.548	0.461	< .001	1.000	0.090	0.004	< .001	< .001	0.755	0.685	< .001	1.000
Lateral orbitofrontal	0.138	0.019	< .001	< .001	4.914	0.086	0.001	2.382	0.125	0.028	< .001	< .001	6.821	0.033	0.002	4.492
Lingual	0.098	0.041	0.016	0.017	11.350	0.003	0.004	9.288	0.111	0.035	0.002	0.002	10.039	0.007	0.003	6.760
Medial orbitofrontal	0.097	0.004	< .001	< .001	1.291	0.524	< .001	1.000	0.111	0.004	< .001	< .001	2.534	0.282	< .001	1.000
Middle temporal	0.150	0.029	< .001	< .001	9.055	0.011	0.002	5.046	0.148	0.024	< .001	< .001	6.937	0.031	0.001	3.508
Paracentral	0.079	0.013	< .001	< .001	2.262	0.323	< .001	1.400	0.083	0.005	< .001	< .001	1.754	0.416	< .001	1.001
Parahippocampal	0.066	0.013	< .001	< .001	3.094	0.213	< .001	1.363	0.071	0.005	< .001	< .001	1.037	0.595	< .001	1.000
Pars opercularis	0.113	0.028	< .001	< .001	8.319	0.016	0.002	4.180	0.077	0.005	< .001	< .001	0.524	0.769	< .001	1.000
Pars orbitalis	0.099	0.005	< .001	< .001	1.983	0.371	< .001	1.004	0.098	0.005	< .001	< .001	1.359	0.507	< .001	1.011
Pars triangularis	0.091	0.005	< .001	< .001	1.593	0.451	< .001	1.002	0.079	0.005	< .001	< .001	1.309	0.520	< .001	1.006
Pericalcarine	0.066	0.010	< .001	< .001	2.629	0.269	< .001	1.161	0.083	0.018	< .001	< .001	3.929	0.140	0.001	2.000
Postcentral	0.095	0.004	< .001	< .001	0.648	0.723	< .001	1.000	0.105	0.015	< .001	< .001	2.970	0.227	< .001	1.662
Posterior cingulate	0.093	0.005	< .001	< .001	0.564	0.754	< .001	1.000	0.111	0.023	< .001	< .001	5.171	0.075	0.001	3.076
Precentral	0.091	0.004	< .001	< .001	0.212	0.899	< .001	1.000	0.094	0.004	< .001	< .001	0.513	0.774	< .001	1.000
Precuneus	0.122	0.022	< .001	< .001	6.262	0.044	0.001	3.025	0.135	0.027	< .001	< .001	10.328	0.006	0.002	4.604
Rostral anterior cingulate	0.109	0.025	< .001	< .001	6.290	0.043	0.001	3.466	0.104	0.019	< .001	< .001	4.261	0.119	0.001	2.135
Rostral middle frontal	0.126	0.017	< .001	< .001	3.866	0.145	< .001	1.971	0.130	0.019	< .001	< .001	4.860	0.088	0.001	2.378
Superior frontal	0.122	0.016	< .001	< .001	3.868	0.145	< .001	1.971	0.147	0.027	< .001	< .001	11.637	0.003	0.002	4.824
Superior parietal	0.108	0.023	< .001	< .001	5.589	0.061	0.001	3.125	0.119	0.027	< .001	< .001	7.744	0.021	0.002	4.167
Superior temporal	0.150	0.023	< .001	< .001	7.701	0.021	0.001	3.362	0.122	0.004	< .001	< .001	1.091	0.580	< .001	1.000
Supramarginal	0.107	0.018	< .001	< .001	4.157	0.125	0.001	2.103	0.088	0.005	< .001	< .001	1.539	0.463	< .001	1.002
Temporal pole	0.074	0.016	< .001	< .001	2.992	0.224	< .001	1.673	0.021	0.023	0.358	0.358	5.629	0.060	0.001	2.634
Transverse temporal	0.080	0.005	< .001	< .001	0.742	0.690	< .001	1.000	0.087	0.007	< .001	< .001	1.815	0.404	< .001	1.068

Table S21 Meta-analysis results for regional *g*-**thickness** associations.

Volume	Left hemisphere								Right hemisphere							
Region	B	*SE*	*p*	FDR Q	Cochrane's *Q*	*p* (*Q*)	I^2	H^2	B	*SE*	*p*	FDR Q	Cochrane's *Q*	*p* (*Q*)	I^2	H^2
Bank ssts	0.043	0.036	0.241	0.299	8.411	0.015	0.003	6.538	0.052	0.044	0.233	0.299	13.068	0.001	0.005	10.048
Caudal anterior cingulate	-0.037	0.022	0.087	0.173	4.902	0.086	0.001	2.374	-0.048	0.016	0.002	0.022	2.644	0.267	< .001	1.533
Caudal middle frontal	0.082	0.042	0.049	0.128	14.837	0.001	0.004	9.711	0.043	0.020	0.035	0.107	4.783	0.091	0.001	2.372
Cuneus	0.012	0.005	0.018	0.090	0.027	0.987	< .001	1.000	0.006	0.005	0.233	0.299	3.003	0.223	< .001	1.000
Entorhinal	0.044	0.025	0.082	0.173	5.366	0.068	0.001	3.071	0.084	0.054	0.123	0.197	25.019	< .001	0.008	14.418
Frontal pole	-0.003	0.005	0.514	0.546	0.640	0.726	< .001	1.000	-0.018	0.005	< .001	0.006	0.203	0.903	< .001	1.000
Fusiform	0.071	0.035	0.043	0.123	12.092	0.002	0.003	6.345	0.082	0.047	0.085	0.173	21.225	< .001	0.006	11.839
Inferior parietal	0.029	0.027	0.280	0.334	5.814	0.055	0.002	4.063	0.051	0.048	0.285	0.334	14.151	0.001	0.006	14.709
Inferior temporal	0.022	0.029	0.450	0.486	6.160	0.046	0.002	4.677	0.050	0.052	0.343	0.382	16.035	< .001	0.008	15.565
Insula	0.039	0.013	0.002	0.022	2.278	0.320	< .001	1.367	0.028	0.021	0.172	0.249	4.848	0.089	0.001	2.364
Isthmus cingulate	-0.019	0.005	< .001	0.004	2.659	0.265	< .001	1.002	-0.009	0.005	0.085	0.173	3.710	0.156	< .001	1.002
Lateral occipital	0.008	0.005	0.127	0.197	0.200	0.905	< .001	1.000	0.026	0.027	0.324	0.367	5.861	0.053	0.002	4.114
Lateral orbitofrontal	0.011	0.005	0.032	0.107	0.412	0.814	< .001	1.000	0.008	0.005	0.126	0.197	0.851	0.654	< .001	1.000
Lingual	0.020	0.005	< .001	0.002	1.523	0.467	< .001	1.000	0.058	0.035	0.098	0.185	13.196	0.001	0.003	6.253
Medial orbitofrontal	-0.003	0.005	0.537	0.561	1.272	0.529	< .001	1.000	-0.013	0.005	0.008	0.055	0.735	0.692	< .001	1.000
Middle temporal	0.052	0.048	0.278	0.334	13.709	0.001	0.006	13.324	0.067	0.048	0.162	0.245	16.539	< .001	0.006	13.519
Paracentral	0.036	0.019	0.061	0.144	4.310	0.116	0.001	2.172	0.023	0.017	0.182	0.258	3.801	0.149	< .001	1.991
Parahippocampal	0.036	0.022	0.104	0.190	5.307	0.070	0.001	2.526	0.045	0.036	0.207	0.282	10.369	0.006	0.003	6.277
Pars opercularis	0.032	0.005	< .001	< .001	2.651	0.266	< .001	1.006	0.043	0.020	0.032	0.107	4.642	0.098	0.001	2.298
Pars orbitalis	0.031	0.023	0.169	0.249	5.066	0.079	0.001	2.780	0.008	0.005	0.126	0.197	1.088	0.581	< .001	1.000
Pars triangularis	0.035	0.029	0.220	0.293	6.337	0.042	0.002	4.359	0.012	0.015	0.417	0.457	3.092	0.213	< .001	1.508
Pericalcarine	-0.010	0.026	0.708	0.720	5.266	0.072	0.001	3.457	-0.012	0.005	0.016	0.090	0.966	0.617	< .001	1.000
Postcentral	0.062	0.038	0.106	0.190	9.713	0.008	0.004	9.042	0.035	0.015	0.018	0.090	3.605	0.165	< .001	1.666
Posterior cingulate	-0.003	0.032	0.921	0.921	6.524	0.038	0.002	4.945	-0.017	0.009	0.063	0.144	2.087	0.352	< .001	1.128
Precentral	0.099	0.044	0.024	0.107	16.601	< .001	0.005	10.861	0.095	0.046	0.040	0.118	19.609	< .001	0.006	11.679
Precuneus	0.034	0.016	0.034	0.107	3.590	0.166	< .001	1.745	0.045	0.027	0.096	0.185	6.706	0.035	0.002	4.258
Rostral anterior cingulate	-0.016	0.005	0.001	0.018	1.078	0.583	< .001	1.000	-0.037	0.013	0.004	0.030	2.430	0.297	< .001	1.296
Rostral middle frontal	0.016	0.005	0.003	0.023	2.093	0.351	< .001	1.011	0.002	0.005	0.709	0.720	0.867	0.648	< .001	1.000
Superior frontal	0.027	0.018	0.127	0.197	4.104	0.128	0.001	2.058	0.017	0.017	0.319	0.367	3.633	0.163	< .001	1.834
Superior parietal	0.028	0.015	0.059	0.144	3.187	0.203	< .001	1.628	0.015	0.013	0.242	0.299	3.232	0.199	< .001	1.460
Superior temporal	0.108	0.049	0.027	0.107	22.151	< .001	0.006	13.875	0.124	0.067	0.064	0.144	36.726	< .001	0.013	26.728
Supramarginal	0.043	0.034	0.204	0.282	7.622	0.022	0.003	6.520	0.064	0.032	0.046	0.126	9.204	0.010	0.002	6.020
Temporal pole	0.069	0.031	0.025	0.107	9.404	0.009	0.002	4.532	0.097	0.045	0.032	0.107	20.614	< .001	0.005	9.896
Transverse temporal	0.022	0.010	0.018	0.090	1.643	0.440	< .001	1.136	0.019	0.012	0.118	0.197	2.537	0.281	< .001	1.286

Table S22 Mean cohort **age moderation r**esults for meta-analysed ***g*-volume** associations.

Volume	Left hemisphere								Right hemisphere							
Region	B	*SE*	*p*	FDR Q	Cochrane's *Q*	*p* (*Q*)	I^2	H^2	B	*SE*	*p*	FDR Q	Cochrane's *Q*	*p* (*Q*)	I^2	H^2
Bank ssts	0.008	0.005	0.126	0.613	4.094	0.043	0.001	4.094	0.006	0.005	0.275	0.825	4.372	0.037	0.001	4.372
Caudal anterior cingulate	-0.003	0.004	0.469	0.825	0.083	0.773	< .001	1.000	< .001	0.004	0.979	0.980	0.533	0.466	< .001	1.000
Caudal middle frontal	0.007	0.004	0.129	0.613	2.754	0.097	0.001	2.754	0.002	0.004	0.544	0.825	1.773	0.183	< .001	1.773
Cuneus	-0.002	0.004	0.660	0.859	0.222	0.637	< .001	1.000	-0.009	0.003	0.009	0.367	0.317	0.573	< .001	1.000
Entorhinal	0.002	0.007	0.784	0.862	8.609	0.003	0.003	8.609	0.003	0.006	0.607	0.825	7.730	0.005	0.002	7.730
Frontal pole	-0.006	0.004	0.131	0.613	0.054	0.817	< .001	1.000	-0.003	0.005	0.585	0.825	2.815	0.093	0.001	2.815
Fusiform	0.007	0.008	0.369	0.825	15.817	< .001	0.005	15.817	0.005	0.008	0.527	0.825	17.784	< .001	0.005	17.784
Inferior parietal	0.004	0.007	0.622	0.830	11.770	0.001	0.004	11.770	0.006	0.009	0.500	0.825	22.150	< .001	0.006	22.150
Inferior temporal	0.012	0.006	0.029	0.561	6.430	0.011	0.002	6.430	0.013	0.006	0.042	0.561	9.455	0.002	0.003	9.455
Insula	-0.002	0.003	0.571	0.825	0.695	0.405	< .001	1.000	-0.008	0.003	0.011	0.367	1.166	0.280	< .001	1.166
Isthmus cingulate	0.002	0.004	0.602	0.825	2.345	0.126	< .001	2.345	0.001	0.004	0.786	0.862	0.380	0.537	< .001	1.000
Lateral occipital	-0.003	0.003	0.317	0.825	0.006	0.939	< .001	1.000	0.004	0.004	0.339	0.825	1.896	0.169	< .001	1.896
Lateral orbitofrontal	0.002	0.005	0.600	0.825	3.976	0.046	0.001	3.976	0.005	0.003	0.116	0.613	0.282	0.595	< .001	1.000
Lingual	0.008	0.006	0.135	0.613	5.634	0.018	0.002	5.634	0.008	0.009	0.374	0.825	17.998	< .001	0.006	17.998
Medial orbitofrontal	-0.001	0.003	0.822	0.874	0.059	0.808	< .001	1.000	0.001	0.003	0.709	0.862	0.815	0.367	< .001	1.000
Middle temporal	0.010	0.007	0.124	0.613	11.062	0.001	0.003	11.062	0.007	0.007	0.329	0.825	11.776	0.001	0.003	11.776
Paracentral	-0.003	0.004	0.520	0.825	1.937	0.164	< .001	1.937	0.001	0.004	0.760	0.862	0.624	0.430	< .001	1.000
Parahippocampal	0.001	0.004	0.746	0.862	1.875	0.171	< .001	1.875	0.006	0.008	0.452	0.825	13.141	< .001	0.004	13.141
Pars opercularis	0.005	0.005	0.308	0.825	5.256	0.022	0.001	5.256	0.001	0.004	0.741	0.862	0.890	0.345	< .001	1.000
Pars orbitalis	0.001	0.003	0.799	0.862	0.190	0.663	< .001	1.000	0.006	0.004	0.134	0.613	1.310	0.252	< .001	1.310
Pars triangularis	0.006	0.003	0.094	0.613	0.268	0.605	< .001	1.000	-0.003	0.004	0.450	0.825	0.001	0.971	< .001	1.000
Pericalcarine	-0.004	0.004	0.306	0.825	0.226	0.635	< .001	1.000	-0.002	0.004	0.590	0.825	2.520	0.112	0.001	2.520
Postcentral	0.008	0.004	0.048	0.561	1.720	0.190	< .001	1.720	0.004	0.005	0.463	0.825	4.712	0.030	0.001	4.712
Posterior cingulate	0.003	0.004	0.473	0.825	0.845	0.358	< .001	1.000	0.007	0.003	0.058	0.561	0.495	0.482	< .001	1.000
Precentral	0.005	0.005	0.325	0.825	4.551	0.033	0.001	4.551	0.006	0.007	0.366	0.825	10.267	0.001	0.003	10.267
Precuneus	0.004	0.006	0.450	0.825	7.412	0.006	0.002	7.412	0.004	0.007	0.517	0.825	11.039	0.001	0.003	11.039
Rostral anterior cingulate	0.004	0.004	0.342	0.825	2.730	0.098	0.001	2.730	< .001	0.005	0.980	0.980	2.962	0.085	0.001	2.962
Rostral middle frontal	0.003	0.004	0.480	0.825	3.868	0.049	0.001	3.868	0.002	0.005	0.669	0.859	4.547	0.033	0.001	4.547
Superior frontal	0.004	0.005	0.400	0.825	4.656	0.031	0.001	4.656	-0.003	0.006	0.586	0.825	7.957	0.005	0.002	7.957
Superior parietal	0.007	0.005	0.156	0.662	4.298	0.038	0.001	4.298	0.007	0.006	0.194	0.778	6.523	0.011	0.002	6.523
Superior temporal	0.003	0.008	0.728	0.862	17.088	< .001	0.005	17.088	0.006	0.006	0.319	0.825	9.840	0.002	0.003	9.840
Supramarginal	0.002	0.005	0.701	0.862	4.242	0.039	0.001	4.242	0.007	0.004	0.054	0.561	1.286	0.257	< .001	1.286
Temporal pole	-0.001	0.005	0.878	0.919	4.755	0.029	0.001	4.755	-0.001	0.004	0.788	0.862	0.061	0.805	< .001	1.000
Transverse temporal	< .001	0.004	0.908	0.936	0.691	0.406	< .001	1.000	0.005	0.005	0.365	0.825	3.933	0.047	0.001	3.933

Table S23 Mean cohort **age moderation r**esults for meta-analysed ***g*-surface area** associations.

Volume	Left hemisphere								Right hemisphere							
Region	B	*SE*	*p*	FDR Q	Cochrane's *Q*	*p* (*Q*)	I^2	H^2	B	*SE*	*p*	FDR Q	Cochrane's *Q*	*p* (*Q*)	I^2	H^2
Bank ssts	0.005	0.004	0.209	0.844	1.792	0.181	< .001	1.792	0.004	0.005	0.446	0.924	4.382	0.036	0.001	4.382
Caudal anterior cingulate	-0.001	0.004	0.741	0.954	1.216	0.270	< .001	1.216	0.003	0.004	0.463	0.924	2.131	0.144	< .001	2.131
Caudal middle frontal	0.003	0.004	0.400	0.924	0.390	0.532	< .001	1.000	0.002	0.004	0.497	0.924	0.892	0.345	< .001	1.000
Cuneus	0.002	0.004	0.550	0.924	2.013	0.156	< .001	2.013	-0.006	0.004	0.074	0.844	0.050	0.824	< .001	1.000
Entorhinal	-0.004	0.004	0.310	0.900	1.658	0.198	< .001	1.658	-0.001	0.004	0.700	0.933	0.013	0.909	< .001	1.000
Frontal pole	-0.006	0.004	0.120	0.844	0.691	0.406	< .001	1.000	-0.003	0.005	0.485	0.924	2.573	0.109	0.001	2.573
Fusiform	0.007	0.006	0.231	0.844	7.212	0.007	0.002	7.212	0.002	0.005	0.684	0.930	5.881	0.015	0.002	5.881
Inferior parietal	< .001	0.007	0.992	0.992	10.393	0.001	0.003	10.393	< .001	0.008	0.992	0.992	14.954	< .001	0.004	14.954
Inferior temporal	0.010	0.006	0.080	0.844	7.277	0.007	0.002	7.277	0.009	0.006	0.146	0.844	8.416	0.004	0.002	8.416
Insula	-0.002	0.003	0.484	0.924	0.703	0.402	< .001	1.000	-0.006	0.003	0.071	0.844	0.440	0.507	< .001	1.000
Isthmus cingulate	< .001	0.003	0.941	0.992	0.927	0.336	< .001	1.000	-0.002	0.003	0.626	0.930	0.263	0.608	< .001	1.000
Lateral occipital	-0.004	0.003	0.214	0.844	0.003	0.953	< .001	1.000	0.001	0.003	0.772	0.955	0.672	0.413	< .001	1.000
Lateral orbitofrontal	< .001	0.005	0.973	0.992	4.755	0.029	0.001	4.755	0.007	0.004	0.090	0.844	2.876	0.090	0.001	2.876
Lingual	0.010	0.005	0.033	0.844	3.896	0.048	0.001	3.896	0.008	0.006	0.152	0.844	6.162	0.013	0.002	6.162
Medial orbitofrontal	0.001	0.004	0.744	0.954	1.200	0.273	< .001	1.200	0.004	0.003	0.243	0.844	1.157	0.282	< .001	1.157
Middle temporal	0.006	0.006	0.304	0.900	7.135	0.008	0.002	7.135	0.004	0.006	0.449	0.924	6.286	0.012	0.002	6.286
Paracentral	-0.001	0.004	0.826	0.986	2.108	0.147	< .001	2.108	0.003	0.004	0.344	0.900	0.858	0.354	< .001	1.000
Parahippocampal	0.006	0.004	0.100	0.844	0.395	0.529	< .001	1.000	< .001	0.004	0.931	0.992	1.031	0.310	< .001	1.031
Pars opercularis	0.005	0.006	0.430	0.924	7.283	0.007	0.002	7.283	0.002	0.004	0.571	0.924	0.203	0.652	< .001	1.000
Pars orbitalis	-0.005	0.003	0.159	0.844	< .001	0.992	< .001	1.000	0.004	0.003	0.244	0.844	0.003	0.956	< .001	1.000
Pars triangularis	0.004	0.004	0.241	0.844	0.218	0.640	< .001	1.000	-0.004	0.004	0.264	0.855	0.062	0.803	< .001	1.000
Pericalcarine	0.004	0.004	0.338	0.900	1.682	0.195	< .001	1.682	0.002	0.005	0.658	0.930	3.804	0.051	0.001	3.804
Postcentral	0.002	0.003	0.562	0.924	0.311	0.577	< .001	1.000	< .001	0.004	0.936	0.992	2.963	0.085	0.001	2.963
Posterior cingulate	0.003	0.004	0.454	0.924	0.003	0.960	< .001	1.000	0.007	0.004	0.062	0.844	1.401	0.236	< .001	1.401
Precentral	-0.002	0.003	0.646	0.930	0.001	0.972	< .001	1.000	< .001	0.003	0.990	0.992	0.513	0.474	< .001	1.000
Precuneus	0.003	0.005	0.567	0.924	5.930	0.015	0.002	5.930	0.003	0.007	0.673	0.930	10.228	0.001	0.003	10.228
Rostral anterior cingulate	0.006	0.005	0.248	0.844	4.283	0.039	0.001	4.283	0.003	0.005	0.527	0.924	3.849	0.050	0.001	3.849
Rostral middle frontal	0.002	0.005	0.646	0.930	3.743	0.053	0.001	3.743	0.001	0.005	0.807	0.980	4.859	0.028	0.001	4.859
Superior frontal	0.002	0.004	0.640	0.930	3.729	0.053	0.001	3.729	-0.004	0.006	0.569	0.924	8.889	0.003	0.002	8.889
Superior parietal	0.006	0.004	0.186	0.844	3.202	0.074	0.001	3.202	0.005	0.006	0.330	0.900	6.190	0.013	0.002	6.190
Superior temporal	-0.001	0.006	0.909	0.992	7.326	0.007	0.002	7.326	< .001	0.003	0.987	0.992	1.091	0.296	< .001	1.091
Supramarginal	-0.001	0.005	0.763	0.955	3.738	0.053	0.001	3.738	0.004	0.003	0.227	0.844	0.077	0.782	< .001	1.000
Temporal pole	-0.001	0.004	0.875	0.992	2.839	0.092	0.001	2.839	0.001	0.006	0.924	0.992	5.381	0.020	0.002	5.381
Transverse temporal	0.003	0.004	0.417	0.924	0.084	0.772	< .001	1.000	0.002	0.004	0.627	0.930	1.604	0.205	< .001	1.604

Table S24 Mean cohort **age moderation r**esults for meta-analysed ***g*-thickness** associations.

Volume	Left hemisphere								Right hemisphere							
Region	B	*SE*	*p*	FDR Q	Cochrane's *Q*	*p* (*Q*)	I^2	H^2	B	*SE*	*p*	FDR Q	Cochrane's *Q*	*p* (*Q*)	I^2	H^2
Bank ssts	0.010	0.005	0.037	0.277	2.596	0.107	0.001	2.596	0.011	0.006	0.088	0.438	6.875	0.009	0.002	6.875
Caudal anterior cingulate	0.001	0.006	0.909	0.936	4.674	0.031	0.002	4.674	0.002	0.005	0.700	0.823	2.285	0.131	0.001	2.285
Caudal middle frontal	0.009	0.007	0.246	0.619	11.898	0.001	0.004	11.898	0.003	0.005	0.615	0.804	4.673	0.031	0.001	4.673
Cuneus	-0.001	0.004	0.874	0.936	0.001	0.970	< .001	1.000	-0.006	0.004	0.102	0.438	0.324	0.569	< .001	1.000
Entorhinal	0.007	0.005	0.124	0.491	2.375	0.123	0.001	2.375	0.011	0.010	0.279	0.637	19.504	< .001	0.008	19.504
Frontal pole	0.002	0.004	0.680	0.823	0.470	0.493	< .001	1.000	0.001	0.004	0.718	0.823	0.073	0.787	< .001	1.000
Fusiform	0.006	0.007	0.399	0.713	10.716	0.001	0.004	10.716	0.009	0.009	0.347	0.673	18.351	< .001	0.007	18.351
Inferior parietal	0.007	0.004	0.076	0.438	2.226	0.136	< .001	2.226	0.012	0.005	0.025	0.277	5.465	0.019	0.001	5.465
Inferior temporal	0.008	0.004	0.021	0.277	0.807	0.369	< .001	1.000	0.013	0.005	0.009	0.277	4.258	0.039	0.001	4.258
Insula	0.001	0.004	0.901	0.936	2.271	0.132	< .001	2.271	< .001	0.005	0.995	0.995	4.669	0.031	0.001	4.669
Isthmus cingulate	0.006	0.004	0.103	0.438	0.001	0.978	< .001	1.000	0.006	0.004	0.086	0.438	0.771	0.380	< .001	1.000
Lateral occipital	0.001	0.004	0.702	0.823	0.054	0.816	< .001	1.000	0.007	0.004	0.093	0.438	2.605	0.107	0.001	2.605
Lateral orbitofrontal	0.002	0.004	0.608	0.804	0.149	0.700	< .001	1.000	-0.001	0.004	0.887	0.936	0.831	0.362	< .001	1.000
Lingual	0.001	0.004	0.726	0.823	1.439	0.230	< .001	1.439	0.005	0.008	0.539	0.781	12.731	< .001	0.005	12.731
Medial orbitofrontal	-0.001	0.004	0.874	0.936	1.258	0.262	< .001	1.258	-0.001	0.004	0.717	0.823	0.604	0.437	< .001	1.000
Middle temporal	0.012	0.005	0.021	0.277	4.688	0.030	0.001	4.688	0.011	0.007	0.130	0.491	11.214	0.001	0.004	11.214
Paracentral	-0.003	0.005	0.519	0.781	2.953	0.086	0.001	2.953	< .001	0.005	0.933	0.946	3.488	0.062	0.001	3.488
Parahippocampal	-0.002	0.005	0.681	0.823	4.521	0.033	0.001	4.521	0.008	0.006	0.215	0.619	7.107	0.008	0.002	7.107
Pars opercularis	0.005	0.004	0.152	0.531	0.603	0.437	< .001	1.000	0.002	0.005	0.761	0.848	4.642	0.031	0.001	4.642
Pars orbitalis	0.006	0.005	0.199	0.619	3.111	0.078	0.001	3.111	0.004	0.004	0.318	0.664	0.090	0.764	< .001	1.000
Pars triangularis	0.008	0.005	0.103	0.438	3.004	0.083	0.001	3.004	0.003	0.005	0.442	0.759	2.556	0.110	0.001	2.556
Pericalcarine	-0.008	0.004	0.030	0.277	0.534	0.465	< .001	1.000	-0.004	0.004	0.326	0.664	< .001	0.990	< .001	1.000
Postcentral	0.010	0.005	0.033	0.277	3.428	0.064	0.001	3.428	0.004	0.004	0.379	0.713	3.017	0.082	0.001	3.017
Posterior cingulate	0.009	0.004	0.015	0.277	0.631	0.427	< .001	1.000	0.004	0.004	0.242	0.619	0.719	0.397	< .001	1.000
Precentral	0.009	0.008	0.231	0.619	12.873	< .001	0.004	12.873	0.009	0.009	0.302	0.662	16.477	< .001	0.006	16.477
Precuneus	0.004	0.005	0.447	0.759	3.174	0.075	0.001	3.174	0.007	0.005	0.211	0.619	4.814	0.028	0.001	4.814
Rostral anterior cingulate	0.004	0.004	0.332	0.664	0.136	0.712	< .001	1.000	0.003	0.004	0.539	0.781	2.068	0.150	< .001	2.068
Rostral middle frontal	0.004	0.003	0.273	0.637	0.893	0.345	< .001	1.000	0.002	0.004	0.593	0.804	0.582	0.446	< .001	1.000
Superior frontal	0.003	0.005	0.554	0.784	3.958	0.047	0.001	3.958	0.003	0.005	0.540	0.781	3.380	0.066	0.001	3.380
Superior parietal	0.002	0.005	0.597	0.804	3.099	0.078	0.001	3.099	0.004	0.004	0.392	0.713	2.657	0.103	0.001	2.657
Superior temporal	0.010	0.009	0.281	0.637	18.605	< .001	0.006	18.605	0.014	0.010	0.156	0.531	25.632	< .001	0.008	25.632
Supramarginal	0.009	0.004	0.012	0.277	1.111	0.292	< .001	1.111	0.007	0.006	0.238	0.619	7.237	0.007	0.002	7.237
Temporal pole	0.005	0.007	0.502	0.781	8.678	0.003	0.003	8.678	0.007	0.010	0.474	0.773	18.940	< .001	0.007	18.940
Transverse temporal	-0.002	0.004	0.668	0.823	1.374	0.241	< .001	1.374	0.003	0.004	0.478	0.773	2.050	0.152	< .001	2.050

Table S25 Quadratic model results for g ~ directional gene component scores.

g ~	Component 1	Component 2
Volume	β = -0.432 *SE* = 0.003), p = .0009	β = -0.559 (*SE* = 0.002), p = 3.55e-06
Surface area	β = -0.356 (*SE* = 0.003), p = .007	β = -0.496 (*SE* = 0.002), p = 5.54e-06
Thickness	β = -0.168 (*SE* = 0.004), p = .212	β = -0.218 (*SE* = 0.003), p = .089

Table S26 Regression results of the association between regional mean cell type profiles and *g*-morphometry betas, after correcting for the two major components of gene expression.

Mean	Cell type	β	*SE*	*t*	*p*	*FDR Q*
Volume	Astrocyte	-0.110	0.050	-2.207	0.031	0.097
Area	Astrocyte	-0.094	0.051	-1.859	0.068	0.141
Thickness	Astrocyte	-0.099	0.051	-1.947	0.056	0.136
Volume	CA1Pyramidal	0.020	0.056	0.356	0.723	0.848
Area	CA1Pyramidal	0.046	0.056	0.814	0.419	0.565
Thickness	CA1Pyramidal	-0.016	0.057	-0.278	0.782	0.848
Volume	Endothelial	-0.183	0.084	-2.186	0.032	0.097
Area	Endothelial	-0.148	0.085	-1.741	0.086	0.146
Thickness	Endothelial	-0.171	0.086	-2.001	0.050	0.134
**Volume**	**Ependymal**	**-0.200**	**0.054**	**-3.668**	**0.000**	**0.007**
Area	Ependymal	-0.143	0.057	-2.492	0.015	0.069
**Thickness**	**Ependymal**	**-0.244**	**0.053**	**-4.626**	**0.000**	**0.001**
Volume	Interneuron	-0.036	0.079	-0.450	0.654	0.803
Area	Interneuron	0.115	0.078	1.469	0.147	0.233
Thickness	Interneuron	-0.138	0.079	-1.745	0.086	0.146
**Volume**	**Microglia**	**-0.155**	**0.054**	**-2.894**	**0.005**	**0.035**
**Area**	**Microglia**	**-0.175**	**0.053**	**-3.322**	**0.001**	**0.013**
Thickness	Microglia	-0.003	0.058	-0.056	0.956	0.981
Volume	Mural	-0.131	0.073	-1.785	0.079	0.146
Area	Mural	-0.070	0.075	-0.935	0.353	0.502
Thickness	Mural	-0.192	0.072	-2.650	0.010	0.055
Volume	Oligodendrocyte	0.107	0.086	1.245	0.218	0.327
Area	Oligodendrocyte	0.002	0.087	0.024	0.981	0.981
Thickness	Oligodendrocyte	0.164	0.086	1.912	0.060	0.136
Volume	S1Pyramidal	0.069	0.099	0.691	0.492	0.632
Area	S1Pyramidal	0.223	0.096	2.325	0.023	0.090
Thickness	S1Pyramidal	-0.028	0.101	-0.274	0.785	0.848

Supplementary Text 1: Meta-analytic mean morphometry profile associations with general and specific patterns of human brain gene expression

While the main analysis focuses on regional *g*-associations, in this supplementary analysis, we provide a complimentary analysis on regional morphometry mean profiles. We calculate meta-analytic regional morphometry means (total N = 40,292[1]) and report correlations with the major components of gene expression. Then, while correcting for these components, we identify specific cell-type-morphometry associations and individual gene-morphometry associations.

Previously, with the virtual histology approach, Paus and colleagues have found associations between regional cortical thickness profiles, and those of astrocytes (r = 0.323, p = 1.68e-03) and CA1 pyramidal cells (r = 0.287, p = 2.40e-04) in a sample of 507 young healthy men[18]. In that study, whilst the correlation for microglia was not statistically significant, it was moderate (r = 0.234, p = .066). Similar results were reported in another study from the same group using a different sample[19]. It should be noted that they used a more stringent two-step consistency check for retaining genes than we used in the current study (this resulted in 2511 genes, as opposed to 8235 retained in the current study). Given that it is these three cell types that have differential loading distributions to baseline on Component 1 in the current study (see supplementary data file), we might predict that controlling for this component would eliminate these apparent cell-specific associations. Taking the present approach, it is

[1] *Note* the UKB sample is slightly larger in this analysis than that used to calculate regional *g*-associations in the main text (UKB N = 39,250, compared to N = 37,840). This is due to slightly different exclusion criteria being used (here, there are fewer participant exclusions as criteria were only based on self-report methods at UKB assessment, and not also official records). This sampling difference is very unlikely to affect the mean results – this is supported by the high correlations in Table SA.

possible to identify cell types that are uniquely associated with regional volume, surface area and thickness, beyond their involvement in major components of cortical gene expression. As in the main analysis, the same approach is extended to the profiles of individual genes.

Results

There were no within-cohort sex differences in the regional mean profiles (for all, r = 1.00). There was also very high consistency in the regional mean profiles between the three cohorts (all $r > .91$, all $p < .0001$, see *Table SA*). The meta-analytic mean results are shown in *Figures SA-D*, and the values are reported in the supplementary data file.

Table SA Cross-cohort correlations for regional mean profiles in volume, surface area and thickness (all $p < .0001$).

Cohort comparison	*r* Volume	*r* Surface area	*r* Thickness
LBC–STRADL	0.998	0.999	0.946
STRADL–UKB	0.999	0.998	0.943
UKB–LBC	0.998	0.998	0.917

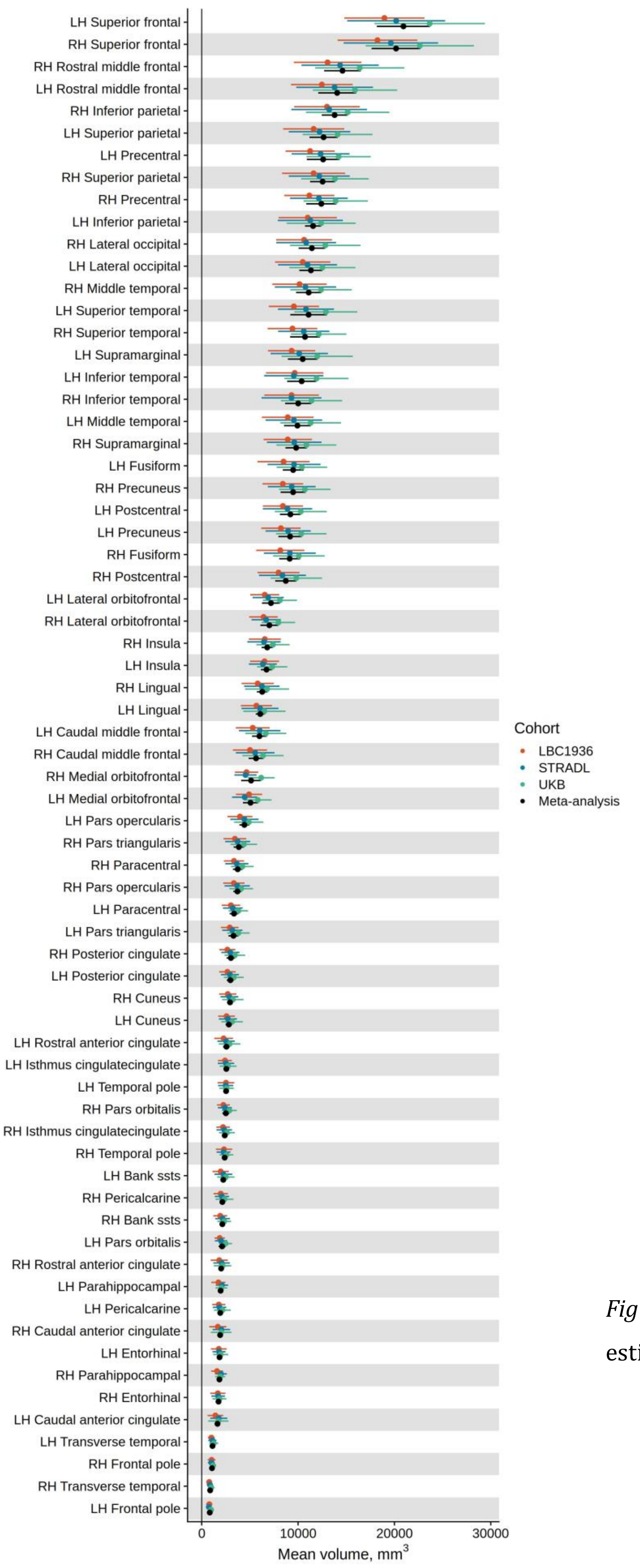

Figure SA Meta-analytic regional estimates of mean volume profiles

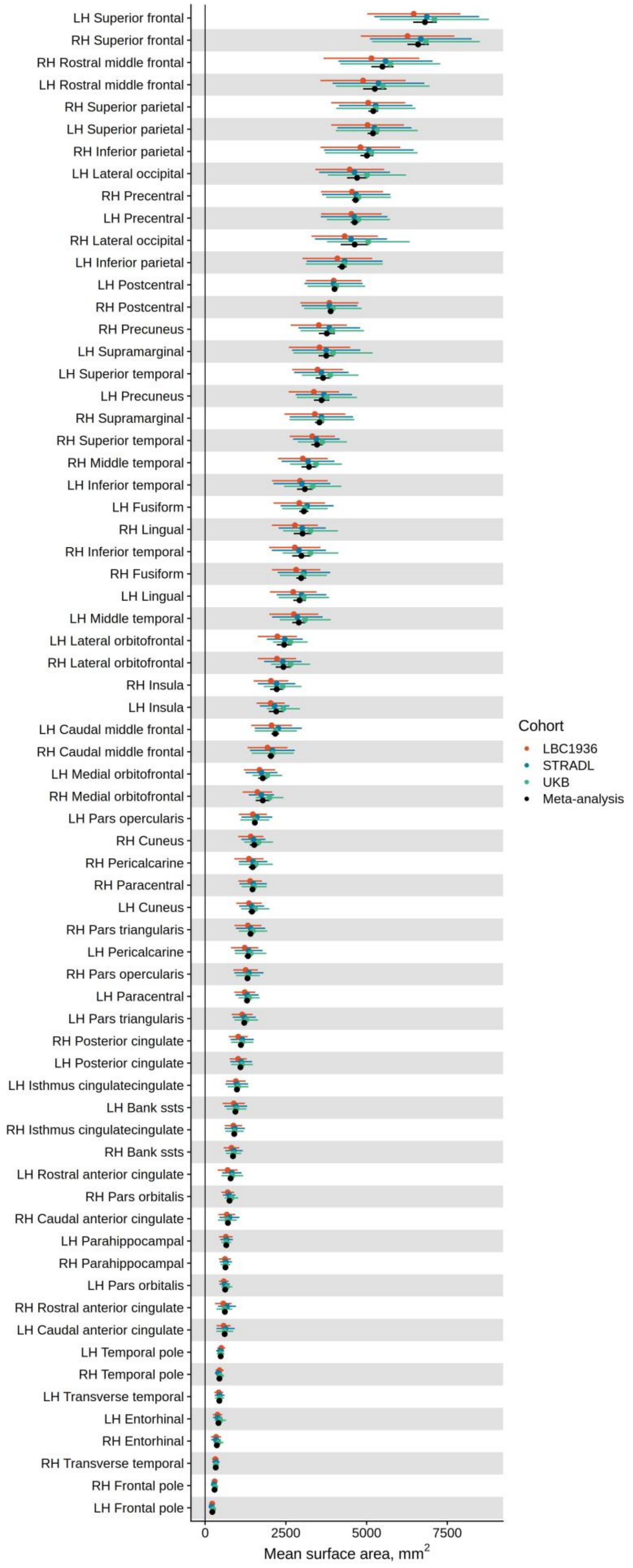

Figure SB Meta-analytic regional estimates of mean surface area profiles

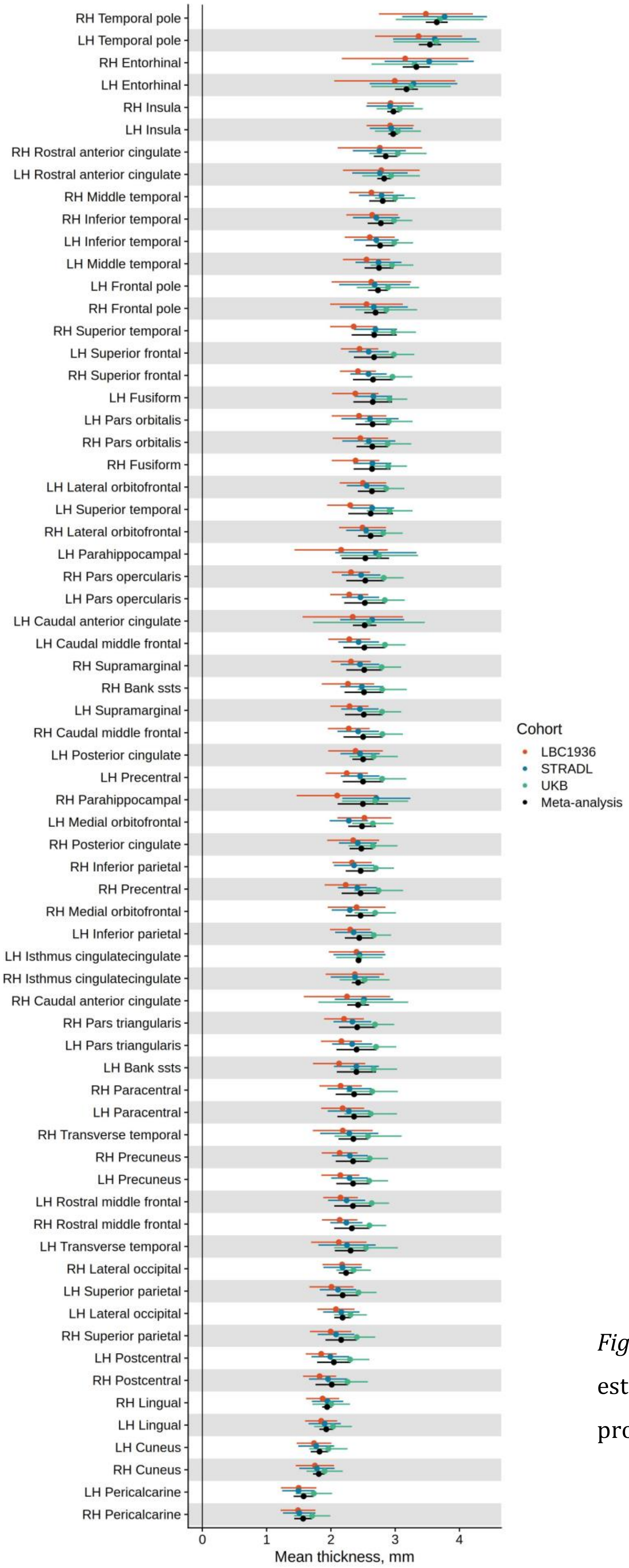

Figure SC Meta-analytic regional estimates of mean thickness profiles

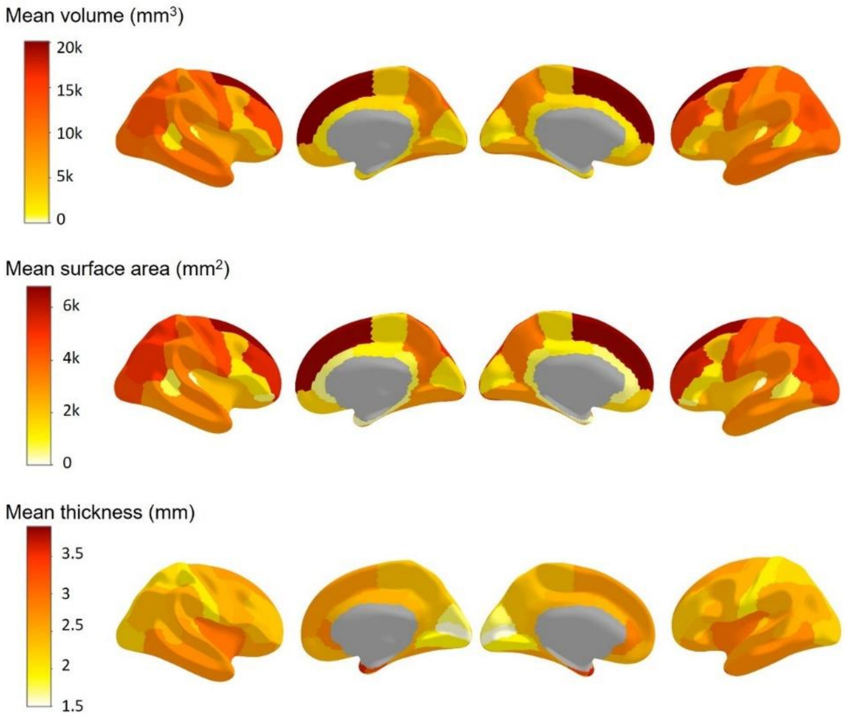

Figure SD Meta-analytic regional mean profiles of volume, surface area and thickness mapped to the cortex.

Correlations between morphometry measures

The regional mean profiles of volume and surface area were closely positively correlated (r = 0.976, p < 2.2e-16), whilst thickness and surface area had a moderate and non-significant negative association (r = -0.226, p = .064), and there was no correlation between volume and thickness (r = -0.055, p = .658).

Associations between regional mean morphometry profiles and *g*-associations

The *g*-associations (reported in the main analysis) were positively associated with their regional mean profiles for all three morphometry measures. The strongest correlation was for volume (r = 0.709, p = 1.35e-11), followed by surface area (r = 0.614, p = 2.58e-08), and then thickness (r = 0.313, p = .009). In other words, regions with stronger *g*-

associations tend to be larger in terms of volume and surface area, and also tend to be thicker.

Associations between regional mean morphometry profiles and the non-absolute component scores or regional overall mean expression profiles

Whilst there are no particularly compelling associations between the regional mean profiles for volume and surface area and the non-absolute component scores, there are strong correlations for thickness (see *Table SB*). The thicker a region is, the more strongly it falls on the regulation side of each gene expression component – cell signalling and modification (r = 0.764, p = 3.67e-14); and transcription factors (r = -0.799, p = 3.132e-16). The regional mean thickness profiles are also moderately associated with the regional gene expression means (r = 0.410, p = .0005) suggesting that simply higher regional levels of gene expression tend to occur in thicker regions.

Table SB Correlations between the regional mean morphometry profiles and the **non-absolute** component scores and mean expression values.

Mean profile	Component 1	Component 2	Mean expression
Volume	r = -0.082, p = .504	r = 0.111, p = .368	r = -0.081, p = .513
Surface area	r = -0.230, p = .059	r = 0.245, p = .044	r = -0.144, p = .243
Thickness	r = 0.764, p = 3.68e-14	r = -0.799, p = 3.13e-16	r = 0.410, p = .0005

Associations between the regional mean morphometry profiles and the absolute component scores

Turning to the absolute component scores, the regions that are most important for defining the two major components of gene expression tend to be moderately smaller in terms of volume and surface area and slightly less thick (see *Table SC*).

Table SC Correlations between the regional mean morphometry measures and the **absolute** component scores and mean expression values.

Mean profile	Component 1	Component 2
Volume	r = -0.369, p = .002	r = -0.471, p = 5.083e-05
Surface area	r = -0.288, p = .017	r = -0.401, p = .0007
Thickness	r = -0.255, p = .036	r = -0.162, p = .187

Associations between regional mean morphometry profiles and specific cell types

Then, correcting for the regional component profiles, we tested for associations between the regional morphometry means and the mean profiles for each of the 9 cell types. The only associations for which FDR $Q < .05$ were for g-volume and g-surface area with ependymal cells: g-volume: β = -0.210, *SE* = 0.054, $p < .001$, g-surface area: β = -0.207, *SE* = 0.056, $p < .001$), see *Table SD*. These associations were small-to-moderate and negative, suggesting that ependymal cells have lower expression levels in larger cortical structures.

Table SD Regression results of the association between regional mean cell type profiles and regional morphometry means, after correcting for the two major components of gene expression.

Mean	Cell type	β	*SE*	*t*	*p*	*FDR Q*
Volume	Astrocyte	-0.620	0.292	-2.125	0.037	0.253
Area	Astrocyte	-0.094	0.052	-1.789	0.078	0.286
Thickness	Astrocyte	-0.050	0.103	-0.488	0.627	0.709
Volume	CA1Pyramidal	-0.027	0.057	-0.479	0.634	0.709
Area	CA1Pyramidal	-0.048	0.058	-0.829	0.410	0.673
Thickness	CA1Pyramidal	0.157	0.111	1.414	0.162	0.318
Volume	Endothelial	-0.122	0.086	-1.418	0.161	0.318
Area	Endothelial	-0.130	0.088	-1.473	0.146	0.318
Thickness	Endothelial	-0.078	0.173	-0.450	0.654	0.709
**Volume**	**Ependymal**	**-0.210**	**0.054**	**-3.871**	**< .001**	**0.007**
**Area**	**Ependymal**	**-0.207**	**0.056**	**-3.678**	**< .001**	**0.007**
Thickness	Ependymal	-0.292	0.114	-2.565	0.013	0.114
Volume	Interneuron	0.061	0.080	0.766	0.446	0.673
Area	Interneuron	0.052	0.082	0.628	0.532	0.709
Thickness	Interneuron	-0.012	0.159	-0.075	0.940	0.940
Volume	Microglia	-0.102	0.056	-1.818	0.074	0.286
Area	Microglia	-0.101	0.058	-1.751	0.085	0.286
Thickness	Microglia	0.055	0.113	0.488	0.627	0.709
Volume	Mural	-0.036	0.075	-0.475	0.636	0.709
Area	Mural	-0.021	0.078	-0.266	0.791	0.822
Thickness	Mural	-0.201	0.147	-1.365	0.177	0.318
Volume	Oligodendrocyte	0.124	0.086	1.445	0.153	0.318
Area	Oligodendrocyte	0.130	0.089	1.468	0.147	0.318
Thickness	Oligodendrocyte	-0.131	0.172	-0.763	0.448	0.673
Volume	S1Pyramidal	0.171	0.098	1.752	0.085	0.286
Area	S1Pyramidal	0.142	0.102	1.398	0.167	0.318
Thickness	S1Pyramidal	0.089	0.198	0.447	0.656	0.709

Associations between regional mean morphometry profiles and individual genes

There were 639 individual genes that had *FDR Q* < .05 for associations with volume mean profiles, 604 with surface area and 153 with thickness. The full FDR-corrected results of this analysis are in the supplementary data file. 561 genes had *Q* < .05 for both volume and surface area, 6 for both volume and thickness and 3 for surface area and thickness. There were three genes that were significant for all three morphometry measure mean profiles: *CCDC19*, *FSTL4* and *NPTX2* (see *Table SE*).

Table SE Standardised β values for the three individual genes for which *FDR Q* < .05 for all three morphometry measure mean profiles, after correcting for the two major components of gene expression.

Mean profile	*CCDC19*	*FSTL4*	*NPTX2*
Volume	β = -0.412	β = 0.289	β = 0.305
Surface area	β = -0.436	β = 0.264	β = 0.288
Thickness	β = 0.715	β = 0.510	β = 0.561

Brief discussion

In this supplementary analysis, we provide a robust meta-analysis of regional cortical means for three morphometry measures: volume, surface area and thickness. There was very high consistency (all *r* > 0.91) in these regional profiles between three large cohorts, demonstrating their reliability.

Regions that are larger in terms of volume and surface area, and thicker tend positively associated with *g*-associations and tend not to be those that define the two components of gene expression.

We found that regional thickness is moderately positively associated with regional mean expression levels, and that thicker regions fall strongly on the regulation side of both the cell-signalling and modification and transcription factors axes.

We then tested for associations between individual cell type profiles and regional morphometric means, after controlling for the two major components of gene expression. We identified ependymal cells as being associated with the mean volume and surface area beyond the two major components. These associations were moderately negative, suggesting that ependymal cells have lower expression levels in larger cortical structures. As predicted, the previously-reported associations between cortical thickness and astrocytes, microglia and CA1 pyramidal cells were not significant, whilst correcting for the two major components of gene expression. This is likely because thickness has strong correlations with both components (Component 1 r = 0.764, Component 2 r = -0.799), and microglia, astrocytes and CA1 pyramidal cells load differentially onto Component 1 than other genes (see supplementary data file). Therefore, such associations are likely to be best-explained in the context of the two components of gene expression, instead of by the mean expression values of specific cell types alone.

Finally, we calculated unique individual gene-morphometry associations, beyond the two major components. Three genes had significant associations with all three morphometry measures: *CCDC19*, *FSTL4* and *NPTX2*. *CCDC19* has previously been found to suppress cell growth[20]. It has a negative association with both volume and surface area, potentially suggesting that its cell growth suppression is less desirable in regions with that are larger in terms of volume and surface area. Conversely, it has a positive association with regional thickness profiles, indicating that it might suppress cell growth in thicker regions. *FSTL4* is thought to play an important role in regulating neurotrophic factors

which regulate morphological plasticity[21] - this could explain its positive associations with all three morphometry measures – larger structures generally require more morphological plasticity. Finally, *NPTX2* appears to be an especially important gene for regional brain organisation – it is positively associated with all three morphometry measures, and it is positively associated with all three *g*-associations from the main body of the current paper. It is thought to play a critical role in synaptic plasticity and protection, as well as being a key gene in epilepsy, Parkinson's Disease and Alzheimer's Disease[22].

References

---

[1] Wong, A. P., French, L., Leonard, G., Perron, M., Pike, G. B., Richer, L., Veillette, S., Pausova, Z., & Paus, T. (2018). Inter-Regional Variations in Gene Expression and Age-Related Cortical Thinning in the Adolescent Brain. *Cerebral cortex* (*New York, N.Y. : 1991)*, *28*(4), 1272–1281. https://doi.org/10.1093/cercor/bhx040

[2] Yeo, R. A., Ryman, S. G., Pommy, J., Thoma, R. J., & Jung, R. E. General cognitive ability and fluctuating asymmetry of brain surface area. *Intelligence*, *56*, 93-98. https://doi.org/10.1016/j.intell.2016.03.002 (2016).

[3] Markello, R. D. et al. Standardizing workflows in imaging transcriptomics with the abagen toolbox. *eLife*, *10*, e72129. 10.7554/eLife.72129 (2021).

[4] Reitan, R.M. & Wolfson, D. The Halstead–Reitan neuropsycholgical test battery: Therapy and clinical interpretation. Neuropsychological Press, Tucson, AZ. (1985).

[5] Ritchie, K. et al. COGNITO: computerized assessment of information processing. *J Psychol Psychother.* 4(2): 10.4172/2161-0487.1000136 (2014).

[6] Smith, A. Symbol digit modalities test. Western Psychological Services, Los Angeles, CA. (1991).

[7] https://biobank.ctsu.ox.ac.uk/crystal/crystal/docs/Pairs.pdf

[8] Benton A, Hamsher K, & Sivan A. Multilingual aphasia examination. 3rd ed. San Antonio, TX: Psychological Corporation; (1994).

[9] Raven J. C.: Raven's progressive matrices and vocabulary scales. Oxford, England: Oxford Psychologists Press Ltd. (1989).

[10] Wechsler, D. Wechsler Adult Intelligence Scale-Third Edition (WAIS-III). San Antonio: Harcourt Assessment Inc. (1997b).

[11] Wechsler D. 3rd ed. The Psychological Corporation; San Antonio, TX: 1997. Wechsler memory scale

[12] Nelson, H.E. & Wilson, J. **National Adult Reading Test (NART)**, Windsor, UK. (1991).

[13] Wechsler, D. **Wechsler test of adult Reading.** Psychological Corporation, San Antonio, TX. (2001).

[14] Lezak, M.D., Howieson, D.B., Loring, D.W., Hannay, H.J. & Fischer, J.S. **Neuropsychological assessment** (4th ed.), Oxford University Press, New York, NY. (2004)

[15] Smith, A. Symbol digit modalities test. Western Psychological Services, Los Angeles, CA. (1991).

[16] Deary, I. J. et al. The functional anatomy of inspection time: an event-related fMRI study. *NeuroIm*age, *22*(4), 1466–1479. 10.1016/j.neuroimage.2004.03.047 (2004).

[17] Deary, I. J., Der, G., & Ford, G. R**eaction times and intelligence differences: A population-based cohort study.** *Intelligence*, 29 (5), 389-399. 10.1016/S0160-2896(01)00062-9 (2001).

[18] Patel, Y. et al. Virtual histology of multi-modal magnetic resonance imaging of cerebral cortex in young men. *NeuroImage*. 218. 116968. 10.1016/j.neuroimage.2020.116968. (2020).

[19] Shin, J. et al. Cell-Specific Gene-Expression Profiles and Cortical Thickness in the Human Brain. *Cerebral cortex, 28*(9), 3267–3277. https://doi.org/10.1093/cercor/bhx197 (2018).

[20] Liu, Z. et al. Candidate tumour suppressor CCDC19 regulates miR-184 direct targeting of C-Myc thereby suppressing cell growth in non-small cell lung cancers. *Journal of cellular and molecular medicine*, *18*(8), 1667–1679. https://doi.org/10.1111/jcmm.12317 (2014).

[21] Suzuki, R. et al. Enhanced extinction of aversive memories in mice lacking SPARC-related protein containing immunoglobulin domains 1 (SPIG1/FSTL4). *Neurobiology of learning and memory*, *152*, 61–70. https://doi.org/10.1016/j.nlm.2018.05.010 (2018).

[22] Chapman G., Shanmugalingam U. & Smith P.D. The Role of Neuronal Pentraxin 2 (NP2) in Regulating Glutamatergic Signaling and Neuropathology. *Front. Cell. Neurosci*. 13:575. doi: 10.3389/fncel.2019.00575 (2020).
